# BrnQ Branched-Chain Amino Acid Transporters Influence Toxin Production by, but Not Growth of, *Clostridium perfringens* Type A Strain ATCC3624

**DOI:** 10.3390/toxins17040187

**Published:** 2025-04-08

**Authors:** Jihong Li, Iman Mehdizadeh Gohari, Isabella Zhang, Bruce A. McClane

**Affiliations:** Department of Microbiology and Molecular Genetics, School of Medicine, University of Pittsburgh, Pittsburgh, PA 15219, USA; jihongli@pitt.edu (J.L.); imehdiza@pitt.edu (I.M.G.); isz7@pitt.edu (I.Z.)

**Keywords:** *Clostridium perfringens*, BrnQ transporters, alpha toxin, perfringolysin O, regulation

## Abstract

By producing alpha toxin (PLC) and perfringolysin O (PFO), *Clostridium perfringens* type A strains are the most common cause of traumatic gas gangrene. *C. perfringens* cannot synthesize branched-chain amino acids (BCAAs), so BCAA transporters are essential for *C. perfringens* growth and survival. *C. perfringens* type A strain ATCC3624 encodes the BrnQ1, BrnQ2, and BrnQ3 BCAA transporters. RT-PCR analyses showed that, with increasing culture time in TY broth, *brnQ2* and *brnQ3* expression levels remained stable but *brnQ1* expression levels declined. Single null mutants unable to produce one of the BrnQ proteins grew and survived similarly as wild type. However, these mutants all showed altered PLC production, especially in the early culture stage, and those effects were reversible by complementation. Therefore, the presence of BrnQ proteins impacts toxin production levels, even though they are not necessary for growth. Interestingly, a triple mutant that was unable to produce any BrnQ protein also grew similarly as ATCC3624. Since BCAA uptake is essential for *C. perfringens*, this strain must produce another (still to be identified) BCAA transporter.

## 1. Introduction

*Clostridium perfringens* is a Gram-positive, spore-forming anaerobe that is ubiquitously distributed in the environment [1,2]. It also causes enteritis, enterotoxemias, and histotoxic infections (most notably gas gangrene) in both humans and other animals [1,2]. Toxins play a major role in the virulence of this pathogen [3,4]. Currently, *C. perfringens* isolates are classified into seven types (A–G) based upon which of the six typing toxin genes (*plc*, *cpb*, *cpe*, *etx*, *iota*, and *netB*) they carry [5].

The current study used *C. perfringens* strain ATCC3624, which classifies as type A. This strain carries both the *plc* gene encoding alpha toxin (PLC), which has phospholipase C and sphingomyelinase activities, and the *pfoA* gene encoding perfringolysin O (PFO), which is a pore-forming toxin [5,6,7,8]. Type A strains are the primary cause of traumatic gas gangrene (clostridial myonecrosis) in humans [9,10,11,12]. After entry into the body, type A isolates grow in muscle and produce PLC and PFO, as well as many exoenzymes. These toxins and enzymes destroy muscle and soft tissue, causing progressive necrosis. They also enter the circulation, which contributes to multiple organ failure and rapid death [9,10,11,12]. PLC and PFO reportedly have synergistic effects in gas gangrene [13]. Necrosis caused by PLC and PFO during gas gangrene involves an inhibition of the inflammatory response and impaired vascular flow [10,11]. We recently reported that, in vitro, ATCC3624 also upregulates PLC and PFO production in the presence of differentiated C2C12 muscle cells, and this effect liberates nutrients from these host cells to promote growth of this type A strain [6]. This upregulation involves the Agr-like quorum sensing system and both the VirS/VirR and EutV/EutW two-component regulatory systems [6].

Branched-chain amino acids (BCAAs), which include isoleucine, leucine, and valine, are essential for protein synthesis. BCAAs are also precursors for branched-chain fatty acids, which are the major fatty acids of the Gram-positive bacterial cell membrane [14]. However, the BCAA biosynthesis pathway is incomplete in *C. perfringens* [15]. Therefore, this bacterium must acquire BCAAs from the environment or, during infection, from the host. Consequently, BCAA transport is very important for *C. perfringens* growth; i.e., *C. perfringens* cannot grow without BCAA uptake [16,17].

Three *brnQ* BCAA transporter genes are present in the published closed *C. perfringens* genomes [15,18] or the >100 genomes deposited in GenBank, but those transporters have never been studied. However, BCAA transporters have been examined in other bacteria. In *Bacillus subtilis*, BrnQ, BcaP, and BraB are the major transporters for isoleucine, valine, and, possibly, leucine [19]. *Corynebacterium glutamicum* and *Lactobacillus delbrueckii* apparently only produce one BCAA transporter, but other bacteria can make multiple BCAA transporters [20,21]. *S. aureus* has three BCAA transporters, namely BrnQ1, BrnQ2, and BcaP [14,22]. *Lactococcus lactis*, *Streptococcus aureus*, *B. subtilis*, and *Bacillus anthracis* also carry multiple *brnQ* genes encoding BCAA transporters [23]. Some of those *brnQ* genes can contribute to virulence. For example, in *B. anthracis*, three *brnQ* transporters are associated with isoleucine and valine transport, and the gene encoding BrnQ3 also contributes to systemic lethality in a mouse model of anthrax [24].

This study constructed a series of isogenic *brnQ* mutants in ATCC3624 to evaluate the involvement of these transporters in growth, survival, and toxin production in vitro. In the culture conditions used, single mutants unable to express any one of these BCAA transporter genes still exhibited wild-type growth and long-term (24 h) viablility (i.e., survival). However, these mutations did affect toxin production, although the effect varied among the mutants. Surprisingly, a triple null mutant unable to produce any of these BCAA transporters also grew at the same rate as the wild-type strain and transiently produced even more PLC or PFO compared to the wild-type parent. These results support the existence of another BCAA transport pathway in *C. perfringens*.

## 2. Results

### 2.1. Expression of the BrnQ Genes During Growth

As mentioned in the Introduction Section, *C. perfringens* lacks BCAA biosynthesis genes, so this bacterium must acquire BCAAs from the environment. Three annotated BCAA transport genes named *brnQ1*, *brnQ2,* and *brnQ3* are present in the sequenced *C. perfringens* genomes, including three published closed genomes [15,18] and ~100 others deposited in GenBank. Among these strains, the open reading frame sequence of each *brnQ* gene is ~99% conserved. Each of these genes appears to be monocistronic. The three BrnQ proteins encoded by these genes are similar in length; i.e., BrnQ1 consists of 428 amino acids (aa); BrnQ2 has 445 aa, and BrnQ3 is comprised of 424 aa. These three proteins share ~40% sequence identity and ~60% sequence similarity. All three proteins are predicted to have 12 transmembrane domains (Figure S1) (predicted by the TMHMM 2.0 program [TMHMM 2.0—DTU Health Tech—Bioinformatic Services]).

**Figure 1:**
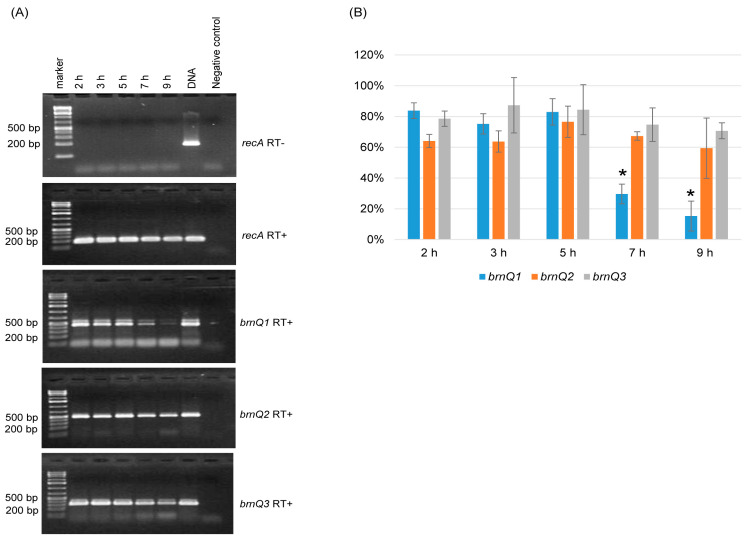
Expression of *brnQ* genes during culture in TY cultures of ATCC3624. (**A**) RT-PCR analyses. The *recA* gene was used as a housekeeping gene, and a sample lacking reverse transcriptase (RT-) was run (top panel) in a PCR reaction with *recA* primers to show the absence of DNA contamination in these RNA samples; *recA* gene RT-PCR (2nd panel) showed that cDNA was present in all samples. Further RT-PCR analyses (bottom three panels) are shown using *brnQ1* primers (for *brnQ1* expression), *brnQ2* primers (for *brnQ2* expression), or *brnQ3* primers (for *brnQ3* expression) for wild-type ATCC3624 cultures at different time points. The expected size of each PCR product is listed in Table 1. The sample with ATCC3624 DNA served as the positive control, and the sample without ATCC3624 DNA served as the negative control. DNA size markers are shown on the left of each gel. The results shown are representative of three repetitions. (**B**) A comparison of the RT-PCR band intensity ratio between the *brnQ* gene vs. the housekeeping *recA* gene is shown using the Image J software. The mean values from three independent experiments are shown. The error bars indicate the S.D. * *p* < 0.05 relative to the 2 h result.

Since *C. perfringens* BrnQ antibodies are not available, this study used RT-PCR analyses to examine the expression of the three *brnQ* genes. An RT-PCR time course study showed that for wild-type ATCC3624 growing at 37 °C in the TY medium, the transcription of *brnQ1*, *brnQ*2, and *brnQ*3 starts by 2 h. However, the expression of *brnQ1* then significantly decreases by 7 h of culture and remains very low at 9 h of culture (Figure 1A). Under the same culture conditions, both *brnQ2* and *brnQ3* expression remains very stable from 2 h to 9 h of culture (Figure 1A). Specifically, a comparison of the RT-PCR band intensity ratio between *brnQ* genes vs. the housekeeping *recA* gene using Image J software (V1.54K, National Institutes of Health, Bethesda, MD, USA) showed that only *brnQ1* gene expression significantly decreases by 7 or 9 h of culture (Figure 1B). As a positive PCR control, ATCC3624’s DNA amplified products using all primers in the absence of RT. The RNA preparations used were not contaminated with DNA since *recA* PCR was performed with those RNA preparations, but in the absence of RT, no product was amplified.

### 2.2. Preparation of BrnQ Single-Gene Mutants and Complementing Strains

To start characterizing the contributions of each BrnQ BCAA transporter to ATCC3624 growth, survival, and toxin production, single *brnQ* null mutant strains were constructed (Figure S2) using the *Clostridium*-modified group II TargeTron^@^ approach [25]. These single null mutant strains were prepared and named brnQ1KO, brnQ2KO, and brnQ3KO. Three complementing strains were also made using the *E. coli* and *C. perfringens* shuttle plasmid pJIR750 [26]. Each of the three complementing plasmids carried one *brnQ* open reading frame (ORF) and its own promoter (Figure S2).

The construction of the three single BrnQ BCAA transporter mutants and the three complementing strains was each confirmed by PCR reactions (Figure S3A). Using the same primers specific for internal *brnQ* ORF sequences, the PCR product amplified from wild-type ATCC3624 DNA was ~900 bp smaller than the PCR products amplified from each null mutant strain due to the targeted insertion of a 900 bp intron in each mutant. Using the same primers, PCR using DNA from the three complementing strains always amplified a product matching the size of the product amplified from the corresponding wild-type *brnQ* gene, and this is indicative of the restored presence of a wild-type copy of this gene in the complementing strain (Figure S3A).

Since these are targeted single *brnQ*-gene mutants, only one intron insertion should be detected in each mutant by intron-specific Southern blot analysis. The Southern blot result in Figure S3B showed that no intron hybridization band occurred using DNA from the wild-type parent and confirmed that only a single intron insertion was present in each single *brnQ* null mutant.

### 2.3. Characterization of the Single BrnQ Mutants and Complementing Strains

RT-PCR was first performed to confirm that, in 2 h TY broth cultures, the *brnQ1* mutant had lost *brnQ1* expression and complementation had restored *brnQ1* expression. Control RT-PCR reactions demonstrated that RNA was not contaminated with DNA, and the quality of the RNA preparation was good. RT-PCR for the expression of the *brnQ1* gene showed that the wild-type parent and complementing strains both express the *brnQ1* transcript. However, no wild-type *brnQ1* transcript was detected using RNA purified from the *brnQ1* null mutant (Figure S4A).

**Figure 2:**
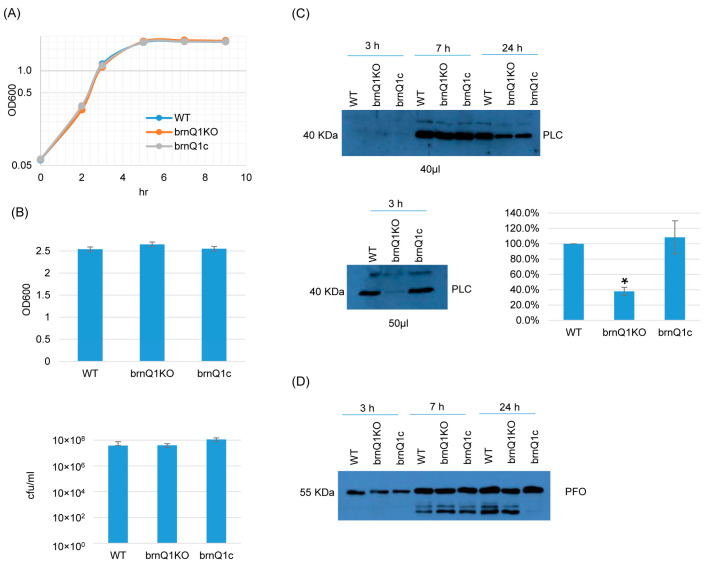
A comparison of post-inoculation changes in culture OD_600_, vegetative cell viability, and PLC or PFO production levels for ATCC3624 (WT), its *brnQ1* null mutant (brnQ1KO), and the complementing strain (brnQ1c) when cultured in a TY medium. (**A**) Post-inoculation changes in the OD_600_ of TY cultures of these strains from 0 to 9 h at 37 °C. The result shown is representative of three repetitions. (**B**) OD_600_ of TY cultures at 24 h at 37 °C (top). The same samples were also used for colony counting of viable vegetative cells (bottom). The mean values from three independent experiments are shown. The error bars indicate the S.D. (**C**) Western blot analyses of PLC level in supernatants of 3, 7, and 24 h culture samples (top panel). The PLC blot was also repeated using 10 µL more of the sample from the same 3 h TY sample, with longer film exposure (bottom left blot), to allow for a better comparison of PLC production between the *brnQ1* null mutant vs. wild-type ATCC3624 or the complementing strain. The size of proteins in kiloDaltons (kDa) is shown on the left. A comparison of the Western blot band intensity ratio is shown between 3 h samples (bottom right graph) using Image J software. The graph shows the mean values from three independent experiments. The error bars indicate the S.D. * *p* < 0.05 relative to wild type. Similar Image J analyses did not detect PLC production differences among these strains in 7 or 24 h samples (Figure S5A). (**D**) Western blot analyses of the PFO level in supernatants of 3, 7, and 24 h culture samples. The results shown are representative of three repetitions. The size of proteins in kiloDaltons (kDa) is shown on the left. Image J analyses did not detect significant differences in PFO production among these strains at any time points (Figure S5A).

The results of growth curve experiments indicated that the wild-type parent, its isogenic *brnQ1* null mutant (brnQ1KO), and the complementing strain (brnQ1c) all grow similarly in a TY medium when cultured up to 9 h at 37 °C (Figure 2A). Extending this analysis further to a 24 h culture time point, culture OD_600_ values and BHI agar plate counting (CFU) results were also similar for these three strains (Figure 2B).

Using supernatants from those same 3, 7, or 24 h TY cultures, PLC and PFO Western blotting were performed. These experiments showed that limited PLC production by these strains began even by 3 h of culture. At this early time point, PLC production by the *brnQ1* null mutant strain was lower compared to its wild-type parent, as better visualized by Western blotting more volume of the same 3 h culture supernatants and exposing the film longer (Figure 2C, bottom panel, left). The complementation of the mutant restored wild-type levels of PLC production. Using Image J software to compare the Western blot band intensity ratio among the wild-type sample, the null mutant and complementing strains confirmed that 3 h cultures of the *brnQ1* null mutant strain makes less PLC compared to the wild-type and complementing strains (Figure 2C). In contrast to those PLC results, no differences in PFO production were noted among the wild-type parent, the *brnQ1* mutant, or the complementing strain at any culture time point (Figure 2D).

**Figure 3:**
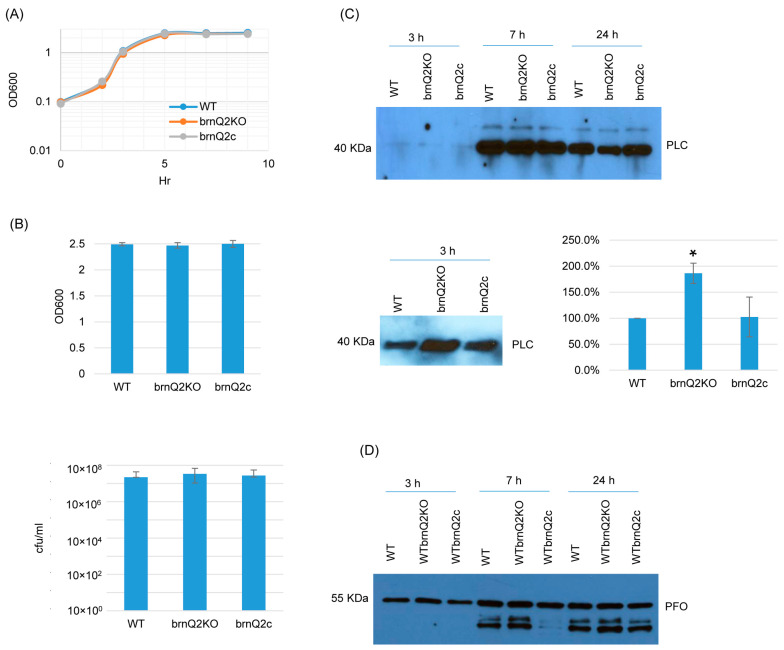
A comparison of post-inoculation changes in culture OD_600_, vegetative cell viability, and PLC or PFO production levels for ATCC3624 (WT), its *brnQ2* null mutant (brnQ2KO), and the complementing strain (brnQ2c) when cultured in TY medium. (**A**) Post-inoculation changes in the OD_600_ of TY cultures of these strains from 0 to 9 h at 37 °C. The result shown is representative of three repetitions. (**B**) The OD_600_ of TY cultures at 24 h at 37 °C (top). The same samples were used for colony counting of viable vegetative cells (bottom). The mean values from three independent experiments are shown. The error bars indicate the S.D. (**C**) Western blot analyses of PLC levels in supernatants of 3, 7, and 24 h culture samples (top panel). The PLC blot was also repeated using 10 µL more of the same 3 h TY samples, with longer film exposure (bottom left blot), to allow for a better comparison of PLC production between the *brnQ2* null mutant vs. wild-type ATCC3624 or the complementing strain. The size of proteins in kiloDaltons (kDa) is shown on the left. A comparison of the Western blot band intensity ratio is shown between 3 h samples (bottom right graph) using Image J software. The mean values from three independent experiments are shown. The error bars indicate the S.D. * *p* < 0.05 relative to the wild type. Similar analyses did not detect PLC production differences among the strains in 7 or 24 h samples (Figure S5B). (**D**) Western blot analyses of PFO levels in supernatants of 3, 7, and 24 h culture samples. The results shown are representative of three repetitions. The size of proteins in kiloDaltons (kDa) is shown at left. Image J software did not detect significant differences in PFO production among the strains at any time points (Figure S5B).

For the *brnQ2* null mutant, RT-PCR confirmed that it does not express *brnQ2,* and complementation restored the expression of this transporter gene (Figure S4B). In TY medium at 37 °C, the *brnQ2* mutant and complementing strain both grew similarly as the wild-type strain (Figure 3A) up to 9 h. Even at a 24 h culture time point, culture OD_600_ values and BHI agar plate counting (CFU) results remained similar for all three strains (Figure 3B). However, in contrast to the *brnQ1* mutant, the *brnQ2* mutant produced more PLC than ATCC3624 after 3 h of culture, and this effect was substantially reversed by complementation (Figure 3C). Using Image J software to compare the Western blot band intensity ratio among the wild-type, null mutant, and complementing strains for 3 h culture confirmed that the *brnQ2* null mutant strain makes more PLC compared to the wild type and complementing strains. By 7 or 24 h of culture, PLC production by the *brnQ2* mutant reverted to wild-type levels (Figure 3C). Similarly, as observed for the *brnQ1* mutant, no differences in PFO production were noted between the *brnQ2* mutant vs. its wild-type parent or complementing strain at any time point (Figure 3D).

**Figure 4:**
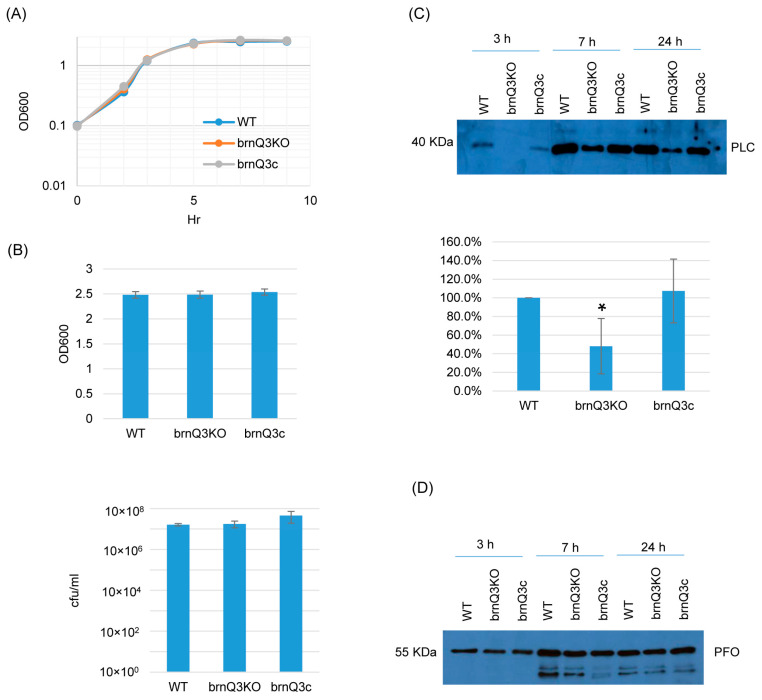
A comparison of the post-inoculation changes in culture OD_600_, vegetative cell viability, and PLC or PFO production levels for ATCC3624 (WT), its *brnQ3* null mutant (brnQ3KO), and the complementing strain (brnQ3c) when cultured in a TY medium. (**A**) Post-inoculation changes in the OD_600_ of TY cultures from 0 to 9 h at 37 °C. The result shown is representative of three repetitions. (**B**) The OD_600_ of TY cultures at 24 h at 37 °C (top). The same samples were also used for colony counting of viable vegetative cells (bottom). The mean values from three independent experiments are shown. The error bars indicate the S.D. (**C**) Western blot analyses of PLC levels in supernatants of 3, 7, and 24 h culture samples (top panel). The size of proteins in kiloDaltons (kDa) is shown on the left. A comparison of the Western blot band intensity ratio is shown between 3 h samples (bottom graph) using Image J software. The mean values from three independent experiments are shown. The error bars indicate the S.D. * *p* < 0.05 relative to the wild type. Similar analyses did not detect PLC production differences among these strains in 7 or 24 h samples (Figure S5C). (**D**) Western blot analyses of PFO levels in supernatants of 3, 7, and 24 h culture samples. The results shown are representative of three repetitions. The size of proteins in kiloDaltons (kDa) is shown on the left. Image J analyses did not detect significant differences in PFO production among these strains at any time points (Figure S5C).

Lastly, RT-PCR showed that the *brnQ3* mutant does not express this transporter gene, and complementation reversed that defect (Figure S4C). The *brnQ3* mutant also grew similarly as its wild-type parent or complementing strain at all time points (Figure 4A,B). For PLC production, the behavior of the *brnQ3* null mutant resembled that of the *brnQ1* null mutant; i.e., this null mutant produced less PLC compared to the wild-type parent or complementing strain, but only for 3 h of culture. Using Image J software to compare the Western blot band intensity ratio among the wild-type, null mutant, and complementing strains for the 3 h culture confirmed that the *brnQ3* null mutant strain makes less PLC compared to the wild-type and complementing strains (Figure 4C). No differences in PFO production were observed between *brnQ3* vs. wild-type ATCC3624 or the complementing strain at any tested time point (Figure 4D).

### 2.4. Expression of Each BrnQ Gene Affects the Expression of the Other BrnQ Genes

**Figure 5:**
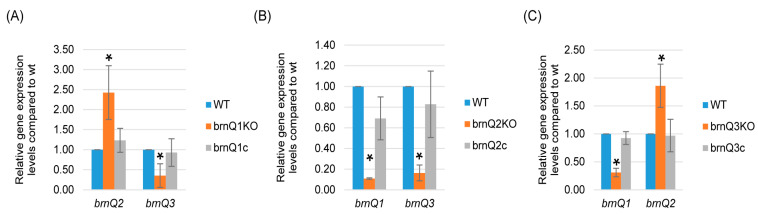
A comparison of expression of the *brnQ* genes by wild-type ATCC3624, its isogenic *brnQ* null mutants, and complementing strains. ATCC3624, the single *brnQ* null mutants, or complementing strains were inoculated into TY broth and then incubated anaerobically for 2 h at 37 °C. Bacteria were collected and pelleted via centrifugation. Total RNA was extracted from the pellets, and cDNA was made for RT-qPCR analyses. Average C_T_ values were normalized to the housekeeping *recA* gene, and the fold differences in expression were calculated using the comparative C_T_ method (2^−ΔΔC_T_^). The mean values from three independent experiments are shown. The error bars indicate the S.D. (**A**) RT-qPCR for the expression of *brnQ2* or *brnQ3* was performed using cDNA from the wild-type parent, the *brnQ1* null mutant, and the complementing strain after their incubation in a TY medium for 2 h. The mean values from three independent experiments are shown. The error bars indicate the S.D. * *p* < 0.05 relative to the wild type. (**B**) RT-qPCR for the expression of *brnQ1* or *brnQ3* was performed using cDNA from the wild type-parent, the *brnQ2* null mutant, and the complementing strain after their incubation in TY medium for 2 h. The mean values from three independent experiments are shown. The error bars indicate the S.D. * *p* < 0.05 relative to the wild type. (**C**) RT-qPCR for the expression of *brnQ1* or *brnQ2* was performed using cDNA from the wild-type parent, the *brnQ3* null mutant, and the complementing strain after their incubation in TY medium for 2 h. The mean values from three independent experiments are shown. The error bars indicate the S.D. * *p* < 0.05 relative to the wild type.

In *S. aureus* USA300 strains, the mutation of *brnQ2* results in the overexpression of *brnQ1* [14]. Therefore, the current study performed qRT-PCR to evaluate if eliminating the expression of one *C. perfringens brnQ* gene affected the expression of the other two *brnQ* genes. The results, shown in Figure 5A, indicated that the *brnQ1* null mutant exhibits increased *brnQ2* expression but decreased *brnQ3* expression. Results for the *brnQ3* null mutant were similar; i.e., for this mutant, the expression of *brnQ2* increased, but the expression of *brnQ1* decreased (Figure 5C). However, for the *brnQ2* null mutant, both *brnQ1* and *brnQ3* expression levels significantly decreased (Figure 5B). Collectively, these results showed that the expression of any *brnQ* gene impacts the expression of the other two *brnQ* genes, but the nature of this regulatory effect varies among the three *brnQ* genes.

### 2.5. Preparation and Characterization of BrnQ Double Null Mutant Strains

To begin testing for functional redundancy among the BrnQ BCAA transporters for *C. perfringens* growth and toxin production, we prepared three *brnQ* double null mutant strains named brnQ2/Q3DKO, brnQ1/Q3DKO, and brnQ1/Q2DKO. PCR confirmed that the only wild-type *brnQ* gene remaining in brnQ2/Q3DKO was the *brnQ1* gene, the only wild-type *brnQ* gene remaining in brnQ1/Q3DKO was the *brnQ2* gene, and the only wild-type *brnQ* gene remaining in brnQ1/Q2DKO was the *brnQ3* gene (Figure S6A). After curing the mutagenesis plasmid from each double mutant, DNA from the wild-type parent and each *brnQ* double null mutant was subjected to Southern blot analysis using an intron-specific probe. No probe hybridization to wild-type ATCC3624 DNA was detected, but two intron insertions were detected using DNA from each double null mutant (Figure S6B). RT-PCR specific for each *brnQ* gene confirmed that the only *brnQ* gene expressed by brnQ2/Q3DKO was *brnQ1*, the only *brnQ* gene expressed by brnQ1/Q3DKO was *brnQ2,* and the only *brnQ* gene expressed by brnQ1/Q2DKO was *brnQ3* (Figure S6C).

**Figure 6:**
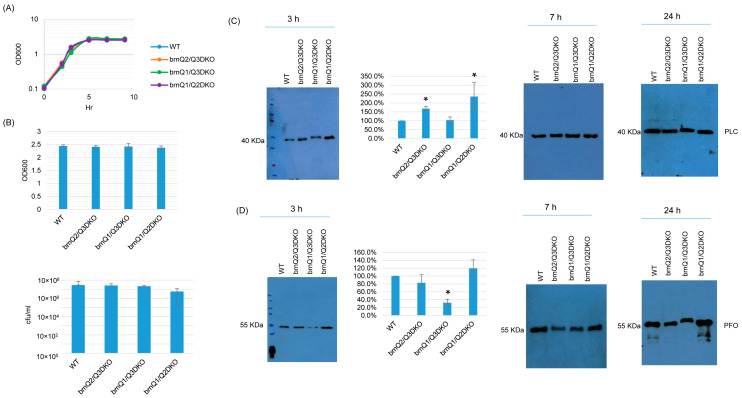
The characterization of double *brnQ* null mutant strains in a TY medium. (**A**) Post-inoculation changes in the OD_600_ of TY cultures from 0 to 9 h at 37 °C. The result shown is representative of three repetitions. (**B**) The OD_600_ of TY cultures at 24 h at 37 °C (top). The same samples were used for colony counting of viable vegetative cells (bottom). The mean values from three independent experiments are shown. The error bars indicate the S.D. (**C**) Western blot analyses of PLC levels in the supernatants of some panel A and panel B samples (3 h, 7 h, and 24 h culture). The results shown are representative of three repetitions. The size of proteins in kiloDaltons (kDa) is shown on the left. A comparison of the Western blot band intensity ratio between 3 h samples (graph) using the Image J software. The mean values from three independent experiments are shown. The error bars indicate the S.D. * *p* < 0.05 relative to the wild type. Similar analyses did not detect any PLC production differences among these strains in 7 or 24 h samples (Figure S7A). (**D**) Western blot analyses of PFO levels in the supernatants of 3, 7, and 24 h culture samples. The results shown are representative of three repetitions. The size of proteins in kiloDaltons (kDa) is shown on the left. A comparison of the Western blot band intensity ratio is shown between 3 h samples (graph) using Image J software. The mean values from three independent experiments are shown. The error bars indicate the S.D. * *p* < 0.05 relative to the wild type. Similar analyses did not detect any PFO production differences among these strains in 7 or 24 h samples (Figure S7B).

TY culture growth curves showed that the double null mutants grew similarly as their wild-type parent (Figure 6A). When culture was extended to 24 h, the OD_600_ and plate count results (CFU) for the double null mutants were also the same as for the wild type (Figure 6B). With respect to toxin production, both PLC and PFO production were impacted by the absence of two *brnQ* genes, but only at 3 h. At that time point, the brnQ2/Q3DKO and brnQ1/Q2DKO mutants produced more PLC compared to their wild-type parent. However, the brnQ1/Q3DKO mutant still produced PLC at wild-type levels. Using Image J software to compare the Western blot band intensity ratio among the wild type and double null mutants for 3 h cultures, the result also confirmed that brnQ2/Q3DKO and brnQ1/Q2DKO make more PLC compared to the wild type (Figure 6C). While there was no effect on PFO production for the single *brnQ* null mutants, PFO production decreased for the brnQ1/Q3DKO strain at 3 h. Using Image J software to compare the Western blot band intensity ratio among the wild type and double null mutants for 3 h cultures, the results also confirmed that only brnQ1/Q3DKO makes less PLC compared to the wild type. (Figure 6D).

**Figure 7:**
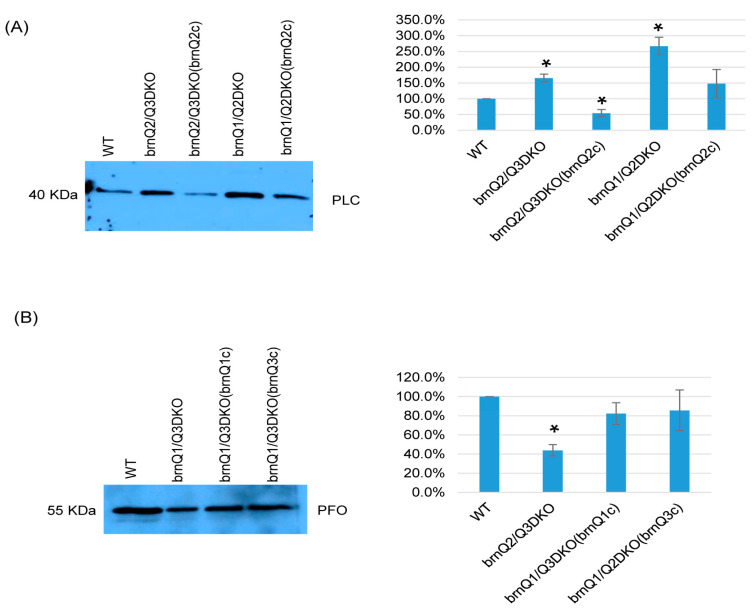
A comparison of PLC or PFO production levels for wild-type ATCC3624, its double null mutants, and the complementing strain when cultured in a TY medium. Western blot analyses of PLC (**A**) and PFO (**B**) levels in supernatants of the wild type, double null mutants, and their complementing strains in the early-stage culture (3 h). The results shown are representative of three repetitions. The size of proteins in kiloDaltons (kDa) is shown on the left. The right figure in panels A and B show the comparison of Western blot intensities determined by Image J. The mean values from three independent experiments are shown. The error bars indicate the S.D. * *p* < 0.05 relative to the wild type.

Results in Figure 6 suggest that, at the early culture stage, BrnQ2 is important for normal PLC production, and BrnQ1 and BrnQ3 together are important for normal PFO production. To confirm these relationships, we complemented the brnQ2/Q3DKO and brnQ1/Q2DKO mutants using a shuttle plasmid containing the *brnQ2* gene. We also prepared complementing strains where the *brnQ1* or *brnQ2* gene were separately transformed into the brnQ1/Q2DKO mutant. Both PCR and RT-PCR results (Figure S8A,B) showed restored *brnQ2* gene expression when the brnQ2/Q3DKO or brnQ1/Q2DKO mutants were complemented with the *brnQ2* gene. Similarly, *brnQ1* or *brnQ3* expression was restored when the brnQ1/Q3DKO mutant was complemented with, respectively, either the *brnQ1* or *brnQ3* gene. Western blot and image J results then revealed that the restoration of *brnQ2* expression via the complementation of brnQ2/Q3DKO or brnQ1/Q2DKO decreased PLC production. After transforming the *brnQ1* or *brnQ3* gene back into the brnQ1/Q3DKO mutant, these complemented strains produced wild-type levels of PFO (Figure 7A,B).

### 2.6. BrnQ Triple Null Mutant Preparation and Characterization

Since BCAA uptake is essential for *C. perfringens* growth and survival [16,17], we explored whether the three BrnQ transporters together are essential for growth and survival but have functional redundancy. If these three transporters are completely redundant but at least one is essential for growth and survival, it should not be possible to construct a triple *brnQ* null mutant. Surprisingly, a triple *brnQ* null mutant strain of ATCC3624 (TKO) was obtained. PCR showed that all three *brnQ* genes contained an intron insertion (Figure S9A). An intron-specific Southern blot indicated that three introns had inserted in the triple null mutant genome (Figure S9B). RT-PCR did not detect the expression of any of these three *BrnQ* genes in this triple null mutant (Figure S9C).

**Figure 8:**
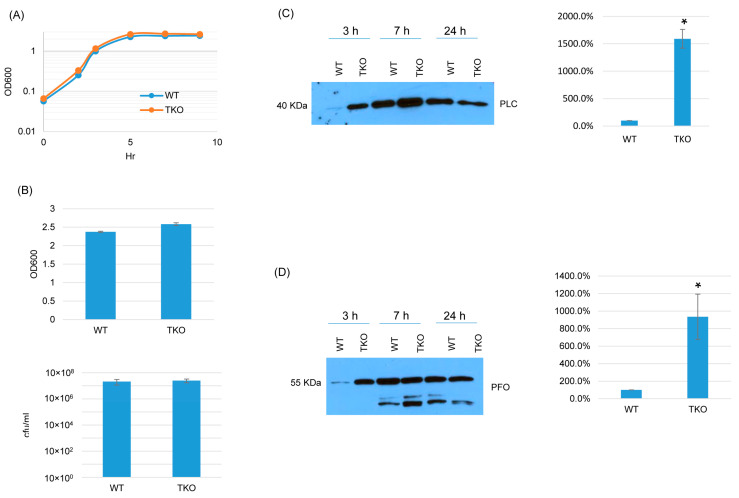
A comparison of post-inoculation changes in culture OD_600_, vegetative cell viability, or PLC and PFO production levels for ATCC3624 (WT) vs. the triple null mutant (TKO) when cultured in a TY medium. (**A**) Post-inoculation changes in the OD_600_ of TY cultures incubated for up to 9 h at 37 °C. The results shown are representative of three repetitions. (**B**) The OD_600_ of TY cultures after 24 h of incubation at 37 °C (top). The same samples were used for colony counting of viable vegetative cells (bottom). The mean values from three independent experiments are shown. The error bars indicate the S.D. (**C**) Western blot analyses of PLC levels in the supernatants of panel A and B samples (3 h, 7 h and 24 h). The results shown are representative of three repetitions. The size of proteins in kiloDaltons (kDa) is shown on the left. A comparison of the Western blot band intensity ratio between 3 h samples (right graph) using Image J software. The mean values from three independent experiments are shown. The error bars indicate the S.D. * *p* < 0.05 relative to the wild type. Similar analyses did not detect any PLC production differences among these strains in 7 or 24 h samples (Figure S10A). (**D**) Western blot analyses of PFO levels in the supernatants of panel A and B samples (3 h, 7 h and 24 h). The results shown are representative of three repetitions. The size of proteins in kiloDaltons (kDa) is shown on the left. The right graph in panel D shows the comparison of the Western blot band intensity ratio between 3 h samples using the Image J software. The mean values from three independent experiments are shown. The error bars indicate the S.D. * *p* < 0.05 relative to the wild type. Similar analyses did not detect any PFO production differences among these strains in 7 or 24 h samples (Figure S10B).

In TY medium, this triple *brnQ* null mutant grew and survived similarly as wild-type ATCC3624 (Figure 8A,B). Interestingly, after the inactivation of all three *brnQ* genes, both PLC and PFO increased compared to wild-type ATCC3624, especially in the early culture stage. Using the Image J software to compare the Western blot band intensity ratio among the wild type and triple null mutants for the 3 h culture, the result also confirmed that TKO makes more PLC and PFO compared to the wild type (Figure 8C,D).

## 3. Discussion

Since *C. perfringens* cannot synthesize BCAAs, the uptake of BCAAs from the environment is essential for its growth and survival [15,16,17]. Despite their importance, the BCAA transporters of *C. perfringens* have not been investigated until now. This study examined the role of BrnQ proteins in *C. perfringens* growth and toxin production since these are important BCAA transporters for several other bacteria. Mutants that are unable to produce one or several of the three BrnQ transporters encoded in the *C. perfringens* genome, namely BrnQ1, BrnQ2 and BrnQ3, were constructed. Notably other pathogenic clostridia, including *Clostridiodes difficile* and *Clostridium botulinum*, also produce BrnQ proteins, but the role of those proteins for the growth of these other clostridial pathogens has not been explored, even though they require BCAA supplementation for growth [27,28,29].

The first finding of the current study is confirmation that all three *brnQ* genes are expressed by *C. perfringens* type A strain ATCC3624. This expression begins during early log phase growth. However, the expression of *brnQ1* later decreased, while *brnQ2* and *brnQ3* expression remained constant into the stationary phase. Furthermore, loss of the expression of one *brnQ* gene was found to affect the expression of the other two *brnQ* genes in *C. perfringens,* although the nature of this effect was variable. The expression of the *brnQ1* gene decreased for both the *brnQ2* and *brnQ3* mutants, and similarly, the expression of *brnQ3* decreased for both the *brnQ1* and *brnQ2* mutants. These effects were all reversible through complementation. In contrast, mutants unable to express either *brnQ1* or *brnQ3* increased *brnQ2* expression, and this effect was also reversible via complementation. Collectively, these results indicate that, in *C. perfringens*, *brnQ2* expression is regulated differently from the expression of the other two *brnQ* genes. This observation is similar to reports with other bacteria where mutating one *brnQ* gene also affected the expression of another *brnQ* gene; e.g., the mutation of *brnQ2* in *S. aureus* resulted in the overexpression of *brnQ1* [14]. To our knowledge, the basis for these regulatory effects of *brnQ* mutations or the expression of other *brnQ* genes is not yet clear for any bacteria.

The current study then used these single, double, and triple *brnQ* null mutants to probe BCAA transporter contributions to ATCC3624 growth and survival in the TY culture. All mutants, even the triple mutant, grew similarly as the wild-type parent. Similarly, there was no difference in 24 h vegetative cell viability (i.e., survival) between the triple *brnQ* mutant vs. the wild type under this culture condition. Determining that these three BCAA transporter genes are not essential for the growth and survival of this strain, at least under these culture conditions, was somewhat surprising since *C. perfringens* cannot synthesize its own BCAAs [15,16,17]. Therefore, these findings imply that there should be another pathway for ATCC3624 to obtain BCAAs from the environment. In other bacteria, this has been established; e.g., *B. subtilis* makes several other permeases, including BcaP and BraB, that (in addition to a BrnQ protein) help mediate BCAA uptake [19]. However, even a triple *brnQ*, *bcaP,* and *braB* null mutant of a *B. subtilis* auxotroph remained viable, indicating that this bacterium still produces at least one additional BCAA transporter [19]. Genes encoding BcaP or BraB homologues were not identifiable in *C. perfringens* genomes. Inspection of *C. perfringens* genomes deposited in GenBank did detect genes encoding 10 annotated amino acid ABC transporters. Those genes are worthy of further investigation [15,18] since the ABC transporter permease LivJHMGF mediates branched-chain amino acid uptake and virulence in *Streptococcus pneumoniae* [30]. Future studies are also needed to distinguish whether, for *C. perfringens*, there is functional redundancy between the BrnQ transporters and the still unidentified non-BrnQ BCAA transporter or if that unidentified transporter is essential for growth.

A final contribution of the current study is demonstrating that the presence of *brnQ* genes impacts toxin production levels by ATCC3624. Previous studies with other pathogens, such as *S. aureus*, hypothesized that the effects of *brnQ* expression on toxin production might explain the virulence attenuation observed for some *brnQ* mutants of those pathogens [22]. However, demonstrating altered toxin production by those *brnQ* null mutants was either not attempted or was inconclusive [24].

For *C. perfringens*, it is notable that the effects of *brnQ* expression on PLC production occur mainly at the early growth stage, a time when all three *brnQ* genes are well expressed. Control of toxin production via the expression of *brnQ* genes does not involve their impact on *C. perfringens’s* growth rate since, as mentioned above, all *brnQ* null mutants grew similarly as the wild-type parent. In addition, while all three single *brnQ* mutants exhibited altered PLC production, their phenotypes varied. Both the single *brnQ1* and *brnQ3* null mutants produced less PLC compared to their wild-type parent in the 3 h culture samples, and those mutants also exhibited significantly more *brnQ2* expression compared to the wild type. In contrast, the *brnQ2* null mutant produced more PLC compared to wild-type ATCC3624 but also showed decreased expression of the *brnQ1* and *brnQ3* genes. Those effects were all reversible through complementation. Double null mutant strains and their complementing strains provided further insights into the effects of *brnQ* gene expression on PLC production. Double mutants that only express *brnQ1* or *brnQ3*, i.e., double mutants lacking *brnQ2* expression, made more PLC at 3 h compared to their wild-type parent. The same phenotype was observed for the triple *brnQ* mutant. Collectively, these results suggest that mutants unable to express *brnQ2*, which also express lower levels of both the *brnQ1* and *brnQ3* genes, sense nutrient stress, i.e., a lower intracellular BCAA pool. In response, *C. perfringens* may try to compensate for this deficit by transiently increasing the production of PLC, which we have shown recently is upregulated in the presence of host cells and can release nutrients (presumably including BCAAs) from those host cells [6].

In contrast to the effects of *brnQ* expression on PLC production, all three single null mutants produced similar PFO levels as wild-type ATCC3624. However, double null mutant strains and their complementing strains showed that the simultaneous loss of expression of both *brnQ1* and *brnQ3* transiently impacts PFO production since a double mutant only expressing *brnQ2* made less PFO than its wild-type parent after 3 h of culture. After complementing one of the inactivated *brnQ* genes in each double mutant, PFO production recovered to wild-type levels. PFO production also transiently increased for the triple mutant, further supporting the importance of the simultaneous expression of *brnQ1* (or *brnQ3*) and *brnQ2* for wild-type levels of PFO production. The profound decreases in expression levels of *brnQ* genes impact PFO production, which also supports the hypothesis that reduced *brnQ* gene expression levels signal *C. perfringens* to nutrient stress, so a transient increase in toxin production is warranted to gain access to more nutrients [6].

The mechanisms behind the regulatory effects of *brnQ* expression on toxin production will require further study. It is not surprising that some *brnQ* mutants showed phenotypic divergence in the production of PLC vs. PFO considering that the regulation of production of these two toxins only partially overlaps [31,32,33]. For some other bacteria, BCAA transporters alter intracellular BCAA levels to regulate the regulatory activity of the BCAA-binding transcriptional regulatory protein CodY, which then impacts toxin production [19,34]. A similar mechanism may also apply to *C. perfringens*. However, a CodY mutant of a *C. perfringens* type D strain did not show altered PLC or PFO activity relative to its parent [35], so the situation could be more complicated in *C. perfringens* and warrants future study.

Lastly, the expression and functional importance of *brnQ* genes may differ in the host versus lab medium. These relationships may even vary among different people. For example, *C. perfringens* is a leading cause of gas gangrene in diabetics [36], who have abnormally high blood BCAA levels [37]. *C. perfringens* growth or virulence in diabetics may be enhanced when those very high levels of BCAAs are taken up by the BCAA transporters of this bacterium. As mentioned, some BCAA transporter mutants of other bacteria can show altered virulence. Our *C. perfringens brnQ* mutants were not tested for their virulence in this initial study for ethical reasons; i.e., it is premature to use animals until the BCAA transporter situation in *C. perfringens* becomes clearer. However, when more information becomes available, future studies should test the most relevant *C. perfringens* BCAA transporter mutants in animal virulence models.

## 4. Materials and Methods

### 4.1. Media and Chemicals

*C. perfringens* was stored in a cooked meat medium (CMM, Oxoid) at −20 °C. *Escherichia coli* DH5α competent cells were kept in as glycerol stocks at −80 °C. The following media were used for the growth of *C. perfringens* wild-type ATCC3624, its isogenic *brnQ* null mutants, and complementing strains: fluid thioglycolate medium (FTG) (Difco Laboratories, Detroit, MI, USA); TY broth (3% tryptic soy broth [Becton-Dickinson Franklin Lakes, NJ, USA], 1% yeast extract [Becton Dickinson], and 0.1% sodium thioglycolate [Sigma-Aldrich, St. Louis, MO, USA]); or TGY broth (TY broth supplemented with 2% glucose [Sigma-Aldrich]). Brain–heart infusion (BHI) agar (Research Products International, Mt. Prospect, IL, USA) plates, with or without 15 μg mL^−1^ chloramphenicol (CM, Sigma-Aldrich), were used to count vegetative cells (CFU) or to select *brnQ* mutants and complementing strains. For anaerobic culture of *C. perfringens*, an MGC AnaeroPack^®^-Anaero (Mitsubishi Gas Chemical CO. INC, Tokyo, Japan) container system was used. To culture DH5α, Luria–Bertani, Miller (LB) broth, or LB agar (Fisher Scientific, Waltham, MA, USA) was used. All bacteria were cultured at 37 °C.

All other chemical reagents used in this study were purchased from Fisher Scientific, Bio-Rad (Hercules, CA, USA), or Sigma Aldrich.

### 4.2. Bacteria Strains, Plasmids, and Primers

The current study used *C. perfringens* type A strain ATCC3624 (ATCC^®^, Manassas VA, USA) and *E. coli* DH5α competent cells [New England Biolabs (NEB), Ipswich, MA, USA]. The plasmid vectors used in this study were the *E. coli*–*C. perfringens* shuttle plasmid pJIR750ai (for making *brnQ* mutants) and pJIR750 (for making *brnQ* complementing strains) [25,26]. The *brnQ* null mutants of ATCC3624 were prepared via the *Clostridium*-modified group II TargeTron^@^ approach [25] using the targeted mutagenesis plasmids pJIR750brnQ1i, pJIR750brnQ2i, and pJIR750brnQ3i. The complementing vectors were pJIR750brnQ1comp, pJIR750brnQ2comp, and pJIR750brnQ3comp. The transformation of ATCC3624 or the *brnQ* null mutant and complementing strains was performed via electroporation and screened using colony PCR [25]. For the preparation of *brnQ* mutants, the intron on the newly constructed *brnQ* mutagenesis plasmids was inserted (in the anti-sense orientation) into the *brnQ1*, *2*, or *3* ORF. The insertion site of each mutant is listed in Table 2 and shown in Figure S2. As previously described [25], the 350 bp intron PCR products amplified using those primers were inserted into pJIR750ai between the *HindIII* and *BsrGI* enzyme sites. To construct double *brnQ* mutants, the *brnQ1* gene was inactivated in the brnQ2KO null mutant to create BrnQ1/2DKO, the *brnQ1* gene was inactivated in the brnQ3KO null mutant to create BrnQ1/Q3DKO, and the *brnQ2* gene was inactivated in the brnQ3KO null mutant to create BrnQ2/Q3DKO. To construct the triple *brnQ* mutant (TKO), we inactivated the *brnQ1* gene in the BrnQ2/Q3DKO double null mutant.

**Table 1:** Primers used in this study.

Primer	Sequence	Size	Purpose
brnQ1-809|810a -IBS	AAAAAAGCTTATAATTATCCTTAAACATCA TAGTGGTGCGCCCAGATAGGGTG	350 bp	*brnQ1* null mutant plasmid (pJIR750brnQ1i)
brnQ1-809|810a -EBS1d	CAGATTGTACAAATGTGGTGATAACAGATA AGTCATAGTGCCTAACTTACCTTTCTTTGT		
brnQ1-809|810a -EBS2	TGAACGCAAGTTTCTAATTTCGGTTATGTTC CGATAGAGGAAAGTGTCT		
EBS universal	CGAAATTAGAAACTTGCGTTCAGTAAAC		
brnQ2-209|210a-IBS	AAAAAAGCTTATAATTATCCTTATGTAACC CTCCTGTGCGCCCAGATAGGGTG	350 bp	*brnQ2* null mutant plasmid (pJIR750brnQ2i)
brnQ2-209|210a-EBS1d	CAGATTGTACAAATGTGGTGATAACAGATA AGTCCCTCCTGATAACTTACCTTTCTTTGT		
brnQ2-209|210a-EBS2	TGAACGCAAGTTTCTAATTTCGGTTTTACAT CGATAGAGGAAAGTGTCT		
EBS universal	CGAAATTAGAAACTTGCGTTCAGTAAAC		
brnQ3- 581|582a-IBS	AAAAAAGCTTATAATTATCCTTAGCTGCCA TTGCAGTGCGCCCAGATAGGGTG	350 bp	*brnQ3* null mutant plasmid (pJIR750brnQ3i)
brnQ3- 581|582a-EBS1d	CAGATTGTACAAATGTGGTGATAACAGATA AGTCATTGCATCTAACTTACCTTTCTTTGT		
brnQ3- 581|582a-EBS2	TGAACGCAAGTTTCTAATTTCGATTGCAGC TCGATAGAGGAAAGTGTCT		
EBS universal	CGAAATTAGAAACTTGCGTTCAGTAAAC		
brnQ1KOF	AGGAACTGGAGGAATTGTAGCA	442 bp (WT) 1342 bp (KO)	*brnQ1* null mutant screening, RT-PCR
brnQ1KOR	GCAGTTTCTAAGGCTGGATCT		
brnQ2KOF	AGTTAATAAAAAAGAAATGGGGTCA	454 bp (WT) 1354 bp (KO)	*brnQ2* null mutant screening, RT-PCR
brnQ2KOR	TGATAAAGATAGCCAATATGCAAC		
brnQ3KOF	ATCTTTGTATTAAAGCCATCAAAAG	291 bp (WT) 1191 bp (KO)	*brnQ3* null mutant screening, RT-PCR
brnQ3KOR	TGCAATAACTGCTGCTTTAATAAG		
brnQ1compF	ATTCgagctcTTAATAGAGGACCTTAAAGGGG TAA (SacI) *	2273 bp	*brnQ1* complementing plasmid (pJIR750brnQ1comp)
brnQ1comR	GCAGgtcgacGGGAAAATATATTCTGAGCTTT TAA (SalI) *		
brnQ2compF	ATTCgagctcAAAAAATACACTTAAGGTTGAC AGC (SacI) *	2030 bp	*brnQ2* complementing plasmid (pJIR750brnQ2comp)
brnQ2comR	GCAGgtcgacTGCTTGTGATTTTGCTTAATTTA TT (SalI) *		
brnQ3compF	ATTCgagctcCAGTTTTTTATAGCGCCTAAGTA AT (SacI) *	2516 bp	*brnQ3* complementing plasmid (pJIR750brnQ3comp)
brnQ3comR	GCAGgtcgacCCCCTCCTACTAAACCAACTAT TAA (SalI) *		
recAF	CTGGTAAAACAACAGTGGCTTT	167 bp	RT-PCR and RT-qPCR for *recA* house-keeping gene [38]
recAR	AGCTTGTTCTCCTGTATCTGGT		
brnQ1qF	GCAGTTTCTAAGGCTGGATCT	122 bp	*brnQ1* RT-qPCR
brnQ1qR	GCAGTCCTTGGCACTACTAAT		
brnQ2qF	CAATAGGACCTGGCTTAGGAATAC	144 bp	*brnQ2* RT-qPCR
brnQ2qR	GCCAATATGCAACTGCGAAA		
brnQ3qF	GCAATCGGAGGACTAGCAATAA	134 bp	*brnQ3* RT-qPCR
brnQ3qR	GCACCTGCATGTCCTAGAATAC		

* Lower letters represent enzyme digestion site.

**Table 2:** *C. perfringens* strains used in this study.

Isolate	Description	Origin
ATCC3624	Wild type	Purchased from ATCC
brnQ1KO	*brnQ1* gene single null mutant strain	This study
brnQ1c	*brnQ1* single null mutant strain complementing the *brnQ1* gene	This study
brnQ2KO	*brnQ2* gene single null mutant strain	This study
brnQ2c	*brnQ2* single null mutant strain complementing the *brnQ2* gene	This study
brnQ3KO	*brnQ3* gene single null mutant strain	This study
brnQ3c	*brnQ3* single null mutant strain complementing the *brnQ3* gene	This study
brnQ2/Q3DKO	*brnQ2* and *brnQ3* genes double null mutant strain	This study
brnQ1/Q3DKO	*brnQ1* and *brnQ3* genes double null mutant strain	This study
brnQ1/Q2DKO	*brnQ1* and *brnQ2* genes double null mutant strain	This study
brnQ2/Q3DKO(brnQ2c)	*brnQ2* and *brnQ3* genes double null mutant strain complemented with the *brnQ2* gene	This study
brnQ1/Q2DKO(brnQ2c)	*brnQ1* and *brnQ2* genes double null mutant strain complemented with the *brnQ2* gene	This study
brnQ1/Q3DKO(brnQ1c)	*brnQ1* and *brnQ2* genes double null mutant strain complemented with the *brnQ1* gene	This study
brnQ1/Q3DKO(brnQ3c)	*brnQ1* and *brnQ2* genes double null mutant strain complemented with the *brnQ3* gene	This study
TKO	*brnQ1*, *brnQ2,* and *brnQ3* genes triple null mutant strain	This study

The primer sequences used for this purpose are listed in Table 1. All RT-PCR and RT-qRCR primers used for assessing the expression of the *brnQ1*, *brnQ2*, and *brnQ3* genes are also listed in Table 1. In this study, the *recA* gene served as a control housekeeping gene for RT-PCR or RT-qPCR experiments [38]. Primers were designed using the software, Vector NTI (version 11.0, 2008), and synthesized using Integrated DNA Technologies (IDT) (Coralville, IO, USA).

### 4.3. C. perfringens DNA Isolation and PCR and Southern Blot Analyses

According to the manufacturer’s instructions, DNA was isolated using the MasterPure Gram-Positive DNA purification kit (Epicenter). Pelleted cells from an aliquot of 3.0 mL of an overnight TGY culture were used to prepare the DNA, and the extracted DNA was then dissolved in ddH_2_O. The annealing temperature for all PCR reactions was 55 °C, with an extension time of 1 min per kb, for a total of 35 cycles. The extension temperature was 72 °C for the DreamTaq Green PCR Master Mix (2X) (Fisher Scientific) and 65 °C for the LongAmp^®^ *Taq* 2X Mix (New England Biolabs).

For Southern blot analyses, a 4 µg aliquot of each DNA was first digested overnight with *PstI* or *BsrGI* at 37 °C (New England Biolabs). Each digested DNA was electrophoresed on a 1% agarose gel before being transferred to a positively charged nylon membrane (Roche, Basel, Switzerland). Those blots were then hybridized with an intron-specific probe that had been prepared with the PCR DIG Probe Synthesis Kit (Roche). All protocols were conducted according to the manufacturer’s instructions.

### 4.4. C. perfringens RNA Isolation and RT-PCR and RT-qPCR Analyses

To obtain RNA from wild-type ATCC3624, bacterial pellets were collected from TY broth cultures grown for 2, 3, 5, 7, and 9 h at 37 °C. A 1 mL aliquot of each culture was removed for optical density measurement at 600 nm (OD_600_) using a Bio-Rad Smart Spectrophotometer. Equivalent OD_600_ values were adjusted to 0.25 for each culture, and those cultures were then pelleted to prepare the RNA. For the RNA preparation of the *brnQ* null mutants and complementing strains, the pellets from two-hour TY cultures were used.

As previously described [35], RNA was extracted using the saturated phenol (Fisher Scientific) method and purified using TRIzol and chloroform (Life technologies [Carlsbad, CA, USA] and Sigma). The purified RNA was then quantified by measuring the absorbance at 260 nm with a Bio-Rad Smart Spectrophotometer. Before being used in RT-PCR or RT-qRCR experiments, an aliquot (50 ng) of every isolated RNA was subjected to PCR (no RT) for *recA* to confirm the purity of the RNA samples.

To perform RT-PCR or RT-qPCR, an aliquot (100 ng) of purified RNA was used for first-strand cDNA synthesis with the Thermo Scientific Maxima First Strand cDNA synthesis kit. Reaction mixtures were incubated in a thermal cycler for 10 min at 25 °C, 30 min at 50 °C, and 5 min at 85 °C to allow for cDNA synthesis. For both RT-PCR and RT-qRCR, *recA* was amplified from samples and utilized as a loading control. RT-PCR used 10–25 ng of cDNA, while RT-qPCR used 5 ng of cDNA, and it was performed as previously described [6]. The Image J software was used to compare the RT-PCR band intensity.

### 4.5. Measurement of the Growth and Survival of ATCC3624, Its BrnQ Null Mutants, and Complementing Strains in a TY Medium

For the analysis of *C. perfringens* growth and long-term viability (survival) in TY, 0.2 mL aliquots from overnight FTG cultures of the wild type, null mutants, or complementing strains were inoculated into 10 mL of TY medium. After overnight culture, a 0.2 mL aliquot from each TY cultures was transferred again to 10 mL of TY medium for another overnight culture. The cultures were incubated at 37 °C; a 1 mL aliquot of the culture was then removed to measure OD_600_ at 0, 2, 3, 5, 7, 9, and 24 h culture times. In addition, to enumerate the survival of the viable vegetative cells of wild-type ATCC3624 and derivative strains, an aliquot of the 24 h TY culture of each sample was serially diluted in sterile PBS (phosphate-buffered saline) and plated onto BHI agar plates. After overnight anaerobic incubation at 37 °C, the colonies on each BHI plate were counted.

### 4.6. Western Blot Analyses of PLC and PFO Production

The TY cultures of the wild-type, null mutant, or complementing strains were adjusted to an equal OD_600_ of ~0.1. A 40 to 50 µL aliquot of supernatants from those cultures was then used for PLC and PFO Western blot analyses, performed as previously described [6]. Image J software was used to compare the Western blot band intensity.

## Data Availability

The authors confirm that the data supporting the findings of this study are available within the article.

## Supplementary Materials

The following supporting information can be downloaded at https://www.mdpi.com/article/10.3390/toxins17040187/s1, Figure S1: Predicted BrnQ1, BrnQ2, and BrnQ3 protein transmembrane domains; Figure S2: Diagram illustrating preparation of *brnQ* null mutants from ATCC3624; Figure S3: PCR and an intron-specific Southern blot verification of each ATCC3624 *brnQ* single null mutant and complementing strain; Figure S4: Characterization of *brnQ1*, *brnQ2* and *brnQ3* expression by RT-PCR for ATCC3624, its *brnQ* single null mutants and complementing strains when cultured in TY medium: Figure S5: Comparison of Western blot intensities as determined by Image J; Figure S6: Verification of ATCC3624 double *brnQ* null mutants by PCR, an intron-specific Southern blot, and RT-PCR analysis; Figure S7: Comparison of Western blot intensities as determined by Image J for wild type (WT) and double null mutant strains; Figure S8: Characterization of complementing strains of *brnQ* double null mutants cultured in TY medium; Figure S9: Confirmation of an ATCC3624 *brnQ* triple null mutant by PCR, an intron-specific Southern blot, and RT-PCR analysis; Figure S10: Comparison of Western blot intensities as determined by Image J for wild type (WT) and triple null mutant strain (TKO).

## Statistical Analyses

All results were compared pairwise against wild-type results using Student’s *t*-test via Excel. Differences were considered significant when the *p* value was less than 0.05.
