# Supplementary material for: BrnQ Branched-Chain Amino Acid Transporters Influence Toxin Production by, but Not Growth of, Clostridium perfringens Type A Strain ATCC3624
Source: Toxins (Basel). 2025 Apr 8;17(4):187. doi: 10.3390/toxins17040187 (PMC12031218; doi:10.3390/toxins17040187)
Supplement: Supplementary file 1 [file toxins-17-00187-s001.zip › toxins-3407990-supplementary.pptx]

## Slide 1
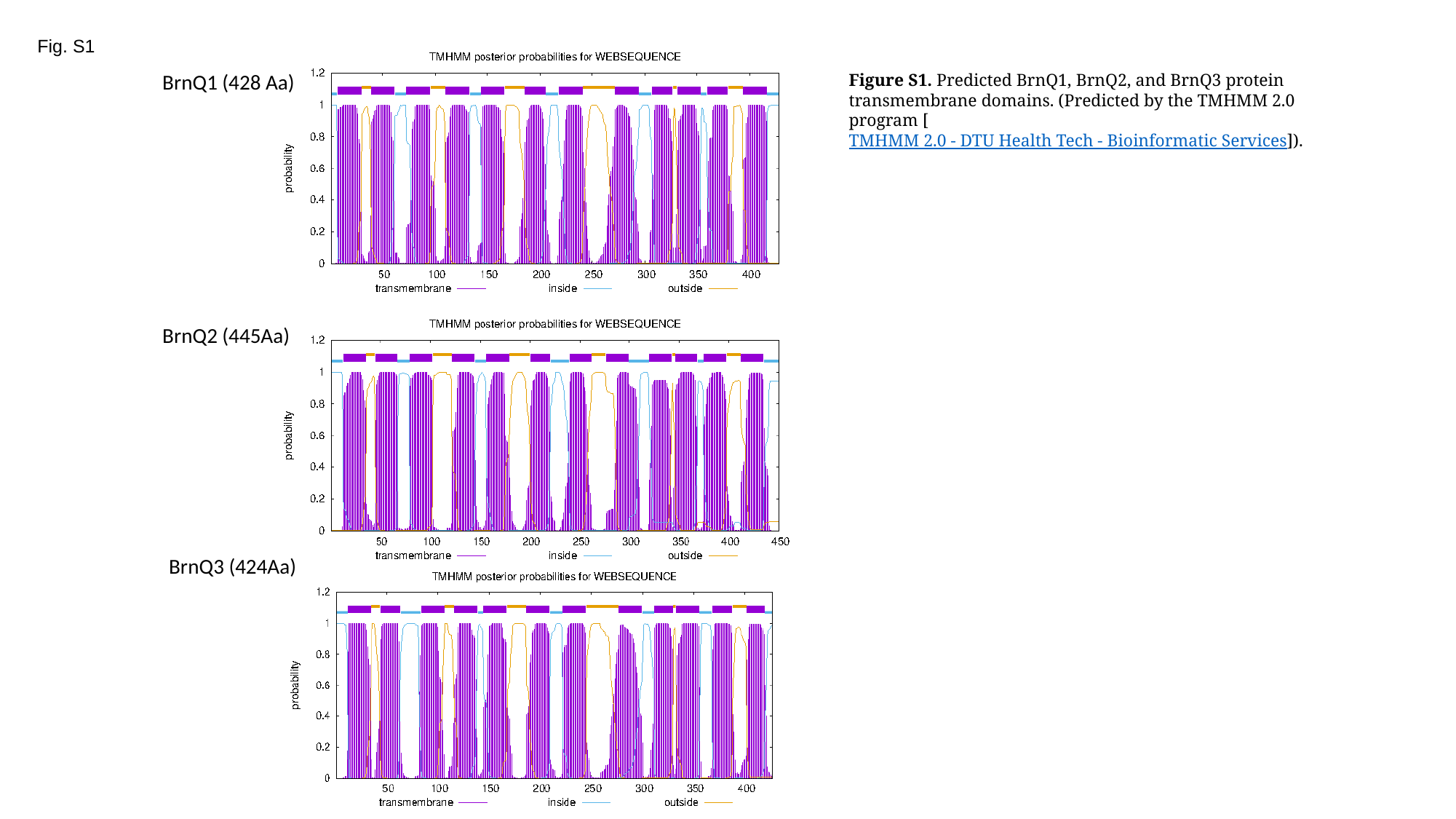

Fig. S1
BrnQ1 (428 Aa)
Figure S1. Predicted BrnQ1, BrnQ2, and BrnQ3 protein transmembrane domains. (Predicted by the TMHMM 2.0 program [TMHMM 2.0 - DTU Health Tech - Bioinformatic Services]).
BrnQ2 (445Aa)
BrnQ3 (424Aa)

## Slide 2
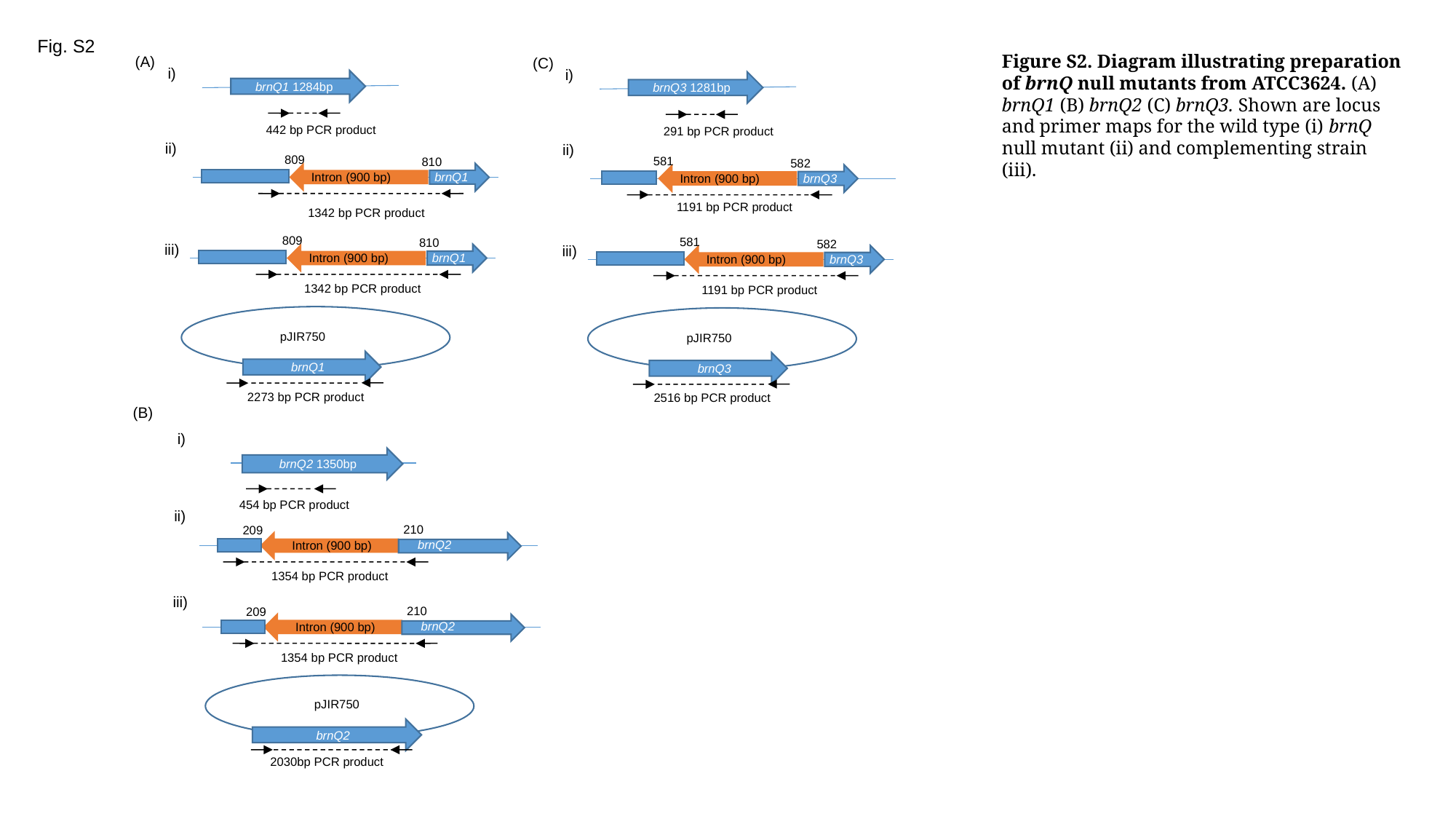

Fig. S2
Figure S2. Diagram illustrating preparation of brnQ null mutants from ATCC3624. (A) brnQ1 (B) brnQ2 (C) brnQ3. Shown are locus and primer maps for the wild type (i) brnQ null mutant (ii) and complementing strain (iii).
(A)
(C)
i)
i)
brnQ1 1284bp
brnQ3 1281bp
442 bp PCR product
291 bp PCR product
ii)
ii)
809
581
810
582
brnQ1
Intron (900 bp)
brnQ3
Intron (900 bp)
1191 bp PCR product
1342 bp PCR product
809
581
810
582
iii)
iii)
brnQ1
Intron (900 bp)
brnQ3
Intron (900 bp)
1342 bp PCR product
1191 bp PCR product
pJIR750
pJIR750
brnQ1
brnQ3
2273 bp PCR product
2516 bp PCR product
(B)
i)
brnQ2 1350bp
454 bp PCR product
ii)
210
209
brnQ2
Intron (900 bp)
1354 bp PCR product
iii)
210
209
brnQ2
Intron (900 bp)
1354 bp PCR product
pJIR750
brnQ2
2030bp PCR product

## Slide 3
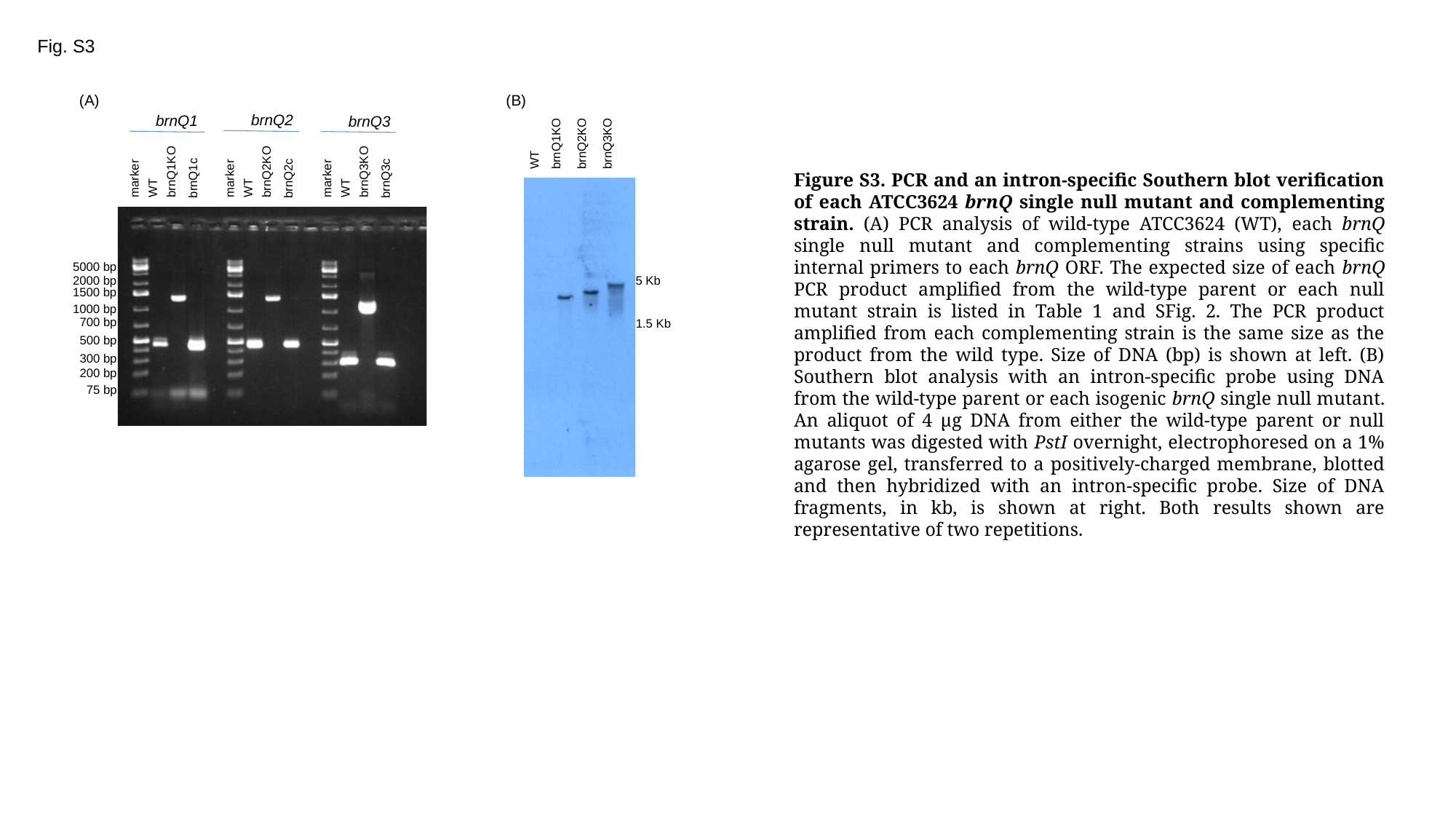

Fig. S3
WT
brnQ1KO
brnQ2KO
brnQ3KO
brnQ1c
brnQ3c
brnQ2c
marker
WT
brnQ1KO
marker
WT
brnQ2KO
marker
WT
brnQ3KO
(A)
(B)
brnQ2
brnQ1
brnQ3
Figure S3. PCR and an intron-specific Southern blot verification of each ATCC3624 brnQ single null mutant and complementing strain. (A) PCR analysis of wild-type ATCC3624 (WT), each brnQ single null mutant and complementing strains using specific internal primers to each brnQ ORF. The expected size of each brnQ PCR product amplified from the wild-type parent or each null mutant strain is listed in Table 1 and SFig. 2. The PCR product amplified from each complementing strain is the same size as the product from the wild type. Size of DNA (bp) is shown at left. (B) Southern blot analysis with an intron-specific probe using DNA from the wild-type parent or each isogenic brnQ single null mutant. An aliquot of 4 µg DNA from either the wild-type parent or null mutants was digested with PstI overnight, electrophoresed on a 1% agarose gel, transferred to a positively-charged membrane, blotted and then hybridized with an intron-specific probe. Size of DNA fragments, in kb, is shown at right. Both results shown are representative of two repetitions.
5000 bp
2000 bp
5 Kb
1500 bp
1000 bp
700 bp
1.5 Kb
500 bp
300 bp
200 bp
75 bp

## Slide 4
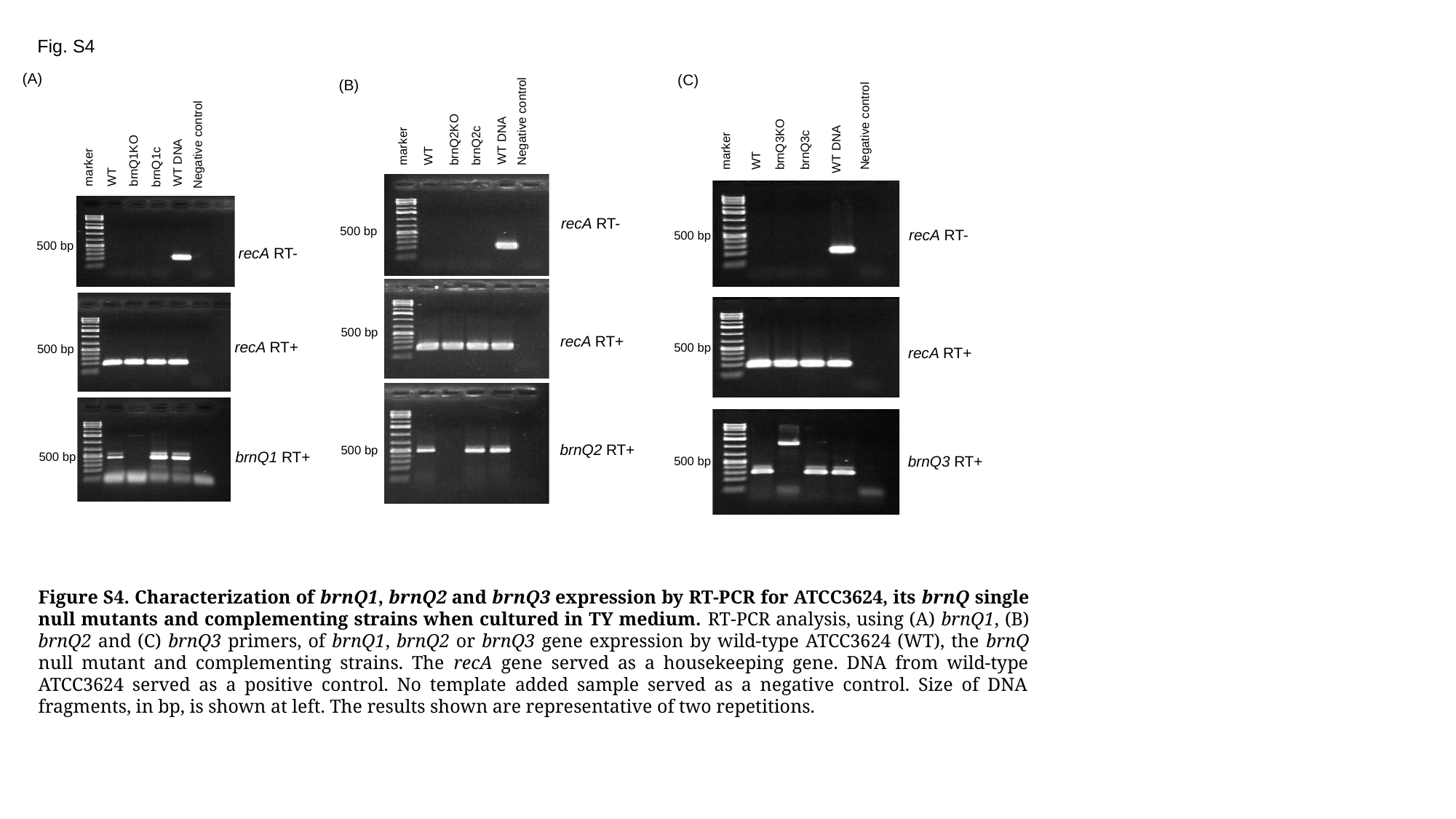

brnQ2c
brnQ3c
Fig. S4
brnQ1c
WT DNA
marker
WT
brnQ2KO
Negative control
marker
WT
brnQ3KO
Negative control
WT DNA
(A)
(C)
(B)
marker
WT
brnQ1KO
WT DNA
Negative control
recA RT-
500 bp
recA RT-
500 bp
500 bp
recA RT-
500 bp
recA RT+
recA RT+
500 bp
500 bp
recA RT+
brnQ2 RT+
500 bp
brnQ1 RT+
500 bp
brnQ3 RT+
500 bp
Figure S4. Characterization of brnQ1, brnQ2 and brnQ3 expression by RT-PCR for ATCC3624, its brnQ single null mutants and complementing strains when cultured in TY medium. RT-PCR analysis, using (A) brnQ1, (B) brnQ2 and (C) brnQ3 primers, of brnQ1, brnQ2 or brnQ3 gene expression by wild-type ATCC3624 (WT), the brnQ null mutant and complementing strains. The recA gene served as a housekeeping gene. DNA from wild-type ATCC3624 served as a positive control. No template added sample served as a negative control. Size of DNA fragments, in bp, is shown at left. The results shown are representative of two repetitions.

## Slide 5
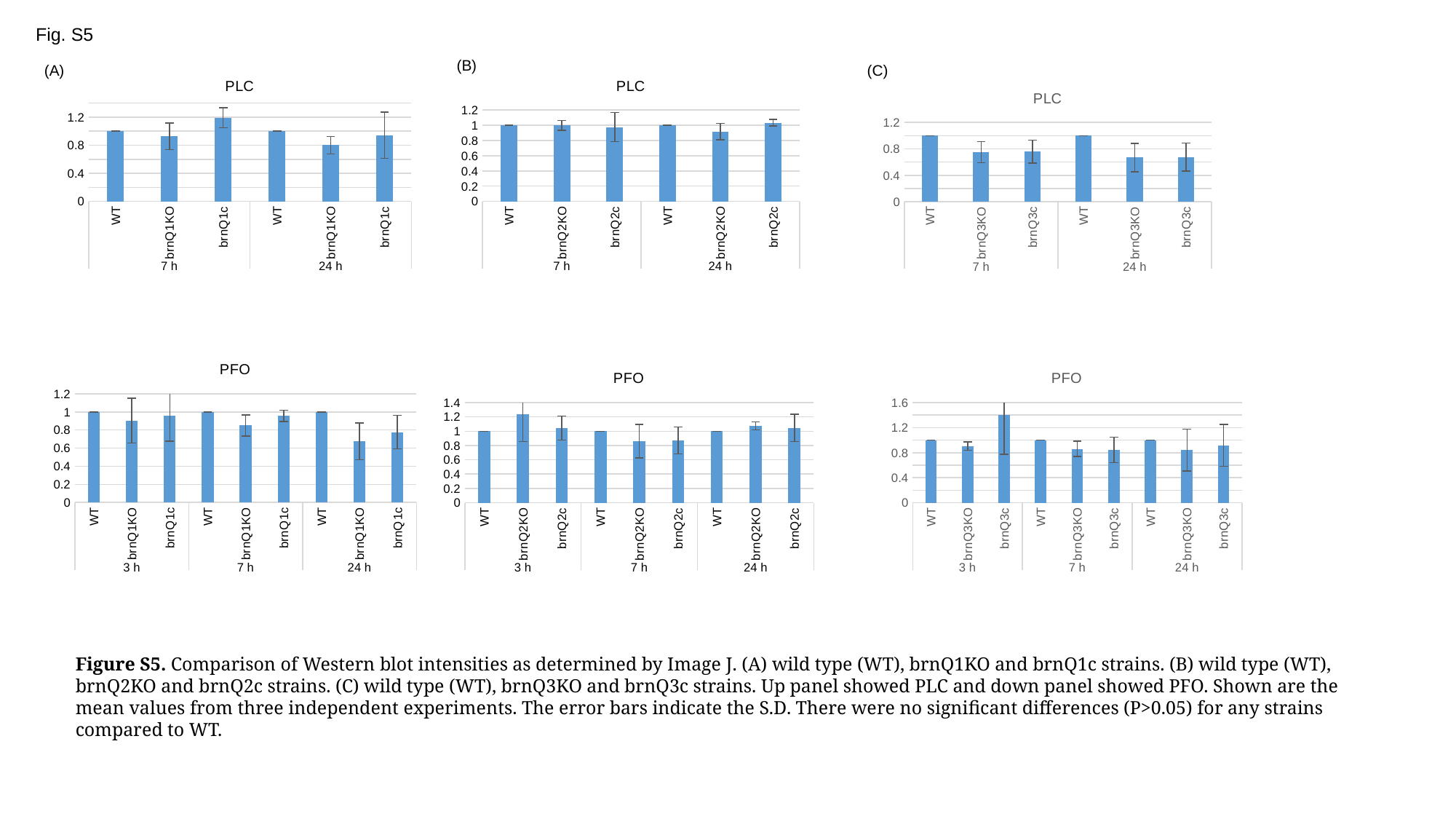

Fig. S5
(B)
(C)
(A)
### Chart: PLC
| Category | |
|---|---|
| WT | 1.0 |
| brnQ1KO | 0.9281349915272837 |
| brnQ1c | 1.1898624632453965 |
| WT | 1.0 |
| brnQ1KO | 0.8009888134852222 |
| brnQ1c | 0.9421615811438918 |
### Chart: PLC
| Category | |
|---|---|
| WT | 1.0 |
| brnQ2KO | 0.9954998410364055 |
| brnQ2c | 0.9747766014166145 |
| WT | 1.0 |
| brnQ2KO | 0.918386085892822 |
| brnQ2c | 1.0321399809767655 |
### Chart: PLC
| Category | |
|---|---|
| WT | 1.0 |
| brnQ3KO | 0.7515873845051076 |
| brnQ3c | 0.7598263694430744 |
| WT | 1.0 |
| brnQ3KO | 0.6677076711419506 |
| brnQ3c | 0.6738421910203064 |
### Chart: PFO
| Category | |
|---|---|
| WT | 1.0 |
| brnQ1KO | 0.9054118857756336 |
| brnQ1c | 0.9574142258297295 |
| WT | 1.0 |
| brnQ1KO | 0.8494922778228541 |
| brnQ1c | 0.9559934868466412 |
| WT | 1.0 |
| brnQ1KO | 0.6760169667466215 |
| brnQ1c | 0.7760481239998519 |
### Chart: PFO
| Category | |
|---|---|
| WT | 1.0 |
| brnQ2KO | 1.2356344422672696 |
| brnQ2c | 1.0430826330612313 |
| WT | 1.0 |
| brnQ2KO | 0.862483314644923 |
| brnQ2c | 0.8715961591975718 |
| WT | 1.0 |
| brnQ2KO | 1.0731766614634972 |
| brnQ2c | 1.0468477258056976 |
### Chart: PFO
| Category | |
|---|---|
| WT | 1.0 |
| brnQ3KO | 0.9040130633638453 |
| brnQ3c | 1.406903703237284 |
| WT | 1.0 |
| brnQ3KO | 0.8611282288429748 |
| brnQ3c | 0.845922071606104 |
| WT | 1.0 |
| brnQ3KO | 0.8415320576119768 |
| brnQ3c | 0.9182960470320186 |Figure S5. Comparison of Western blot intensities as determined by Image J. (A) wild type (WT), brnQ1KO and brnQ1c strains. (B) wild type (WT), brnQ2KO and brnQ2c strains. (C) wild type (WT), brnQ3KO and brnQ3c strains. Up panel showed PLC and down panel showed PFO. Shown are the mean values from three independent experiments. The error bars indicate the S.D. There were no significant differences (P>0.05) for any strains compared to WT.

## Slide 6
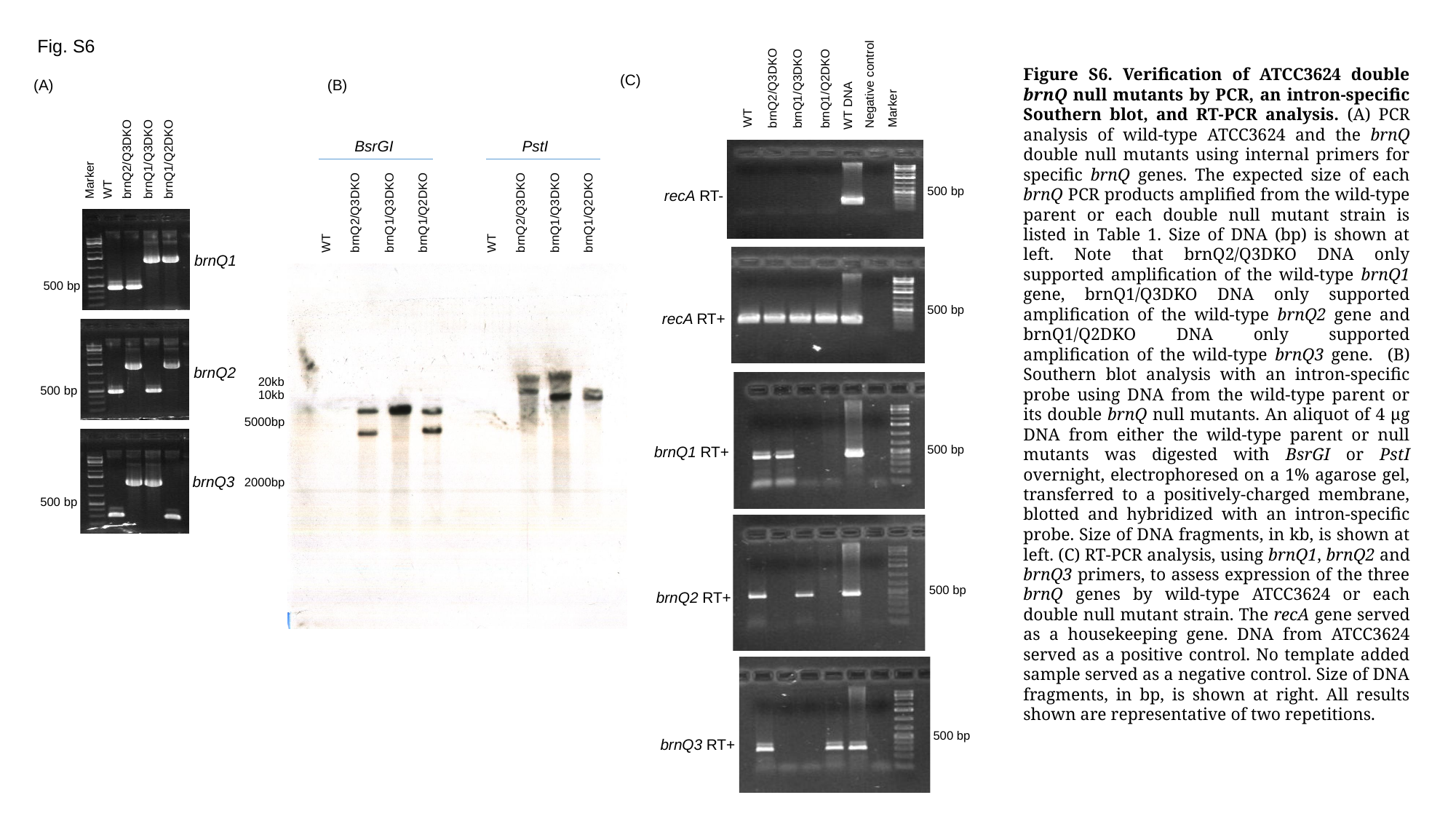

WT
Marker
brnQ2/Q3DKO
brnQ1/Q3DKO
brnQ1/Q2DKO
Negative control
WT DNA
Fig. S6
Figure S6. Verification of ATCC3624 double brnQ null mutants by PCR, an intron-specific Southern blot, and RT-PCR analysis. (A) PCR analysis of wild-type ATCC3624 and the brnQ double null mutants using internal primers for specific brnQ genes. The expected size of each brnQ PCR products amplified from the wild-type parent or each double null mutant strain is listed in Table 1. Size of DNA (bp) is shown at left. Note that brnQ2/Q3DKO DNA only supported amplification of the wild-type brnQ1 gene, brnQ1/Q3DKO DNA only supported amplification of the wild-type brnQ2 gene and brnQ1/Q2DKO DNA only supported amplification of the wild-type brnQ3 gene. (B) Southern blot analysis with an intron-specific probe using DNA from the wild-type parent or its double brnQ null mutants. An aliquot of 4 µg DNA from either the wild-type parent or null mutants was digested with BsrGI or PstI overnight, electrophoresed on a 1% agarose gel, transferred to a positively-charged membrane, blotted and hybridized with an intron-specific probe. Size of DNA fragments, in kb, is shown at left. (C) RT-PCR analysis, using brnQ1, brnQ2 and brnQ3 primers, to assess expression of the three brnQ genes by wild-type ATCC3624 or each double null mutant strain. The recA gene served as a housekeeping gene. DNA from ATCC3624 served as a positive control. No template added sample served as a negative control. Size of DNA fragments, in bp, is shown at right. All results shown are representative of two repetitions.
(C)
(A)
(B)
BsrGI
PstI
WT
brnQ2/Q3DKO
brnQ1/Q3DKO
brnQ1/Q2DKO
WT
brnQ2/Q3DKO
brnQ1/Q3DKO
brnQ1/Q2DKO
20kb
10kb
5000bp
2000bp
Marker
WT
brnQ2/Q3DKO
brnQ1/Q3DKO
brnQ1/Q2DKO
500 bp
recA RT-
brnQ1
500 bp
500 bp
recA RT+
brnQ2
500 bp
500 bp
brnQ1 RT+
brnQ3
500 bp
500 bp
brnQ2 RT+
500 bp
brnQ3 RT+

## Slide 7
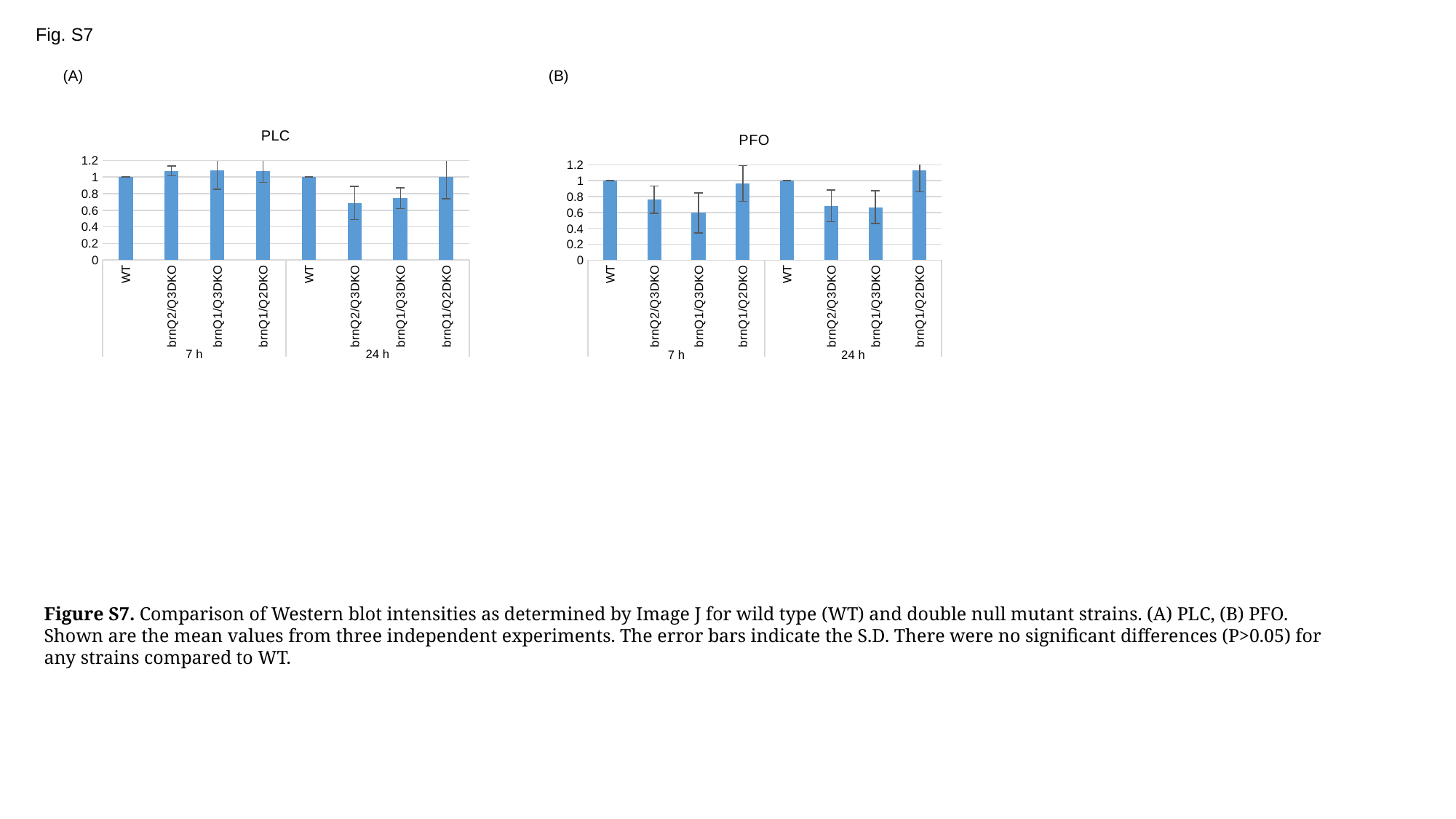

Fig. S7
(B)
(A)
### Chart: PLC
| Category | |
|---|---|
| WT | 1.0 |
| brnQ2/Q3DKO | 1.074757515386761 |
| brnQ1/Q3DKO | 1.078321678256538 |
| brnQ1/Q2DKO | 1.0766358381297676 |
| WT | 1.0 |
| brnQ2/Q3DKO | 0.6883141035360528 |
| brnQ1/Q3DKO | 0.746732539354375 |
| brnQ1/Q2DKO | 1.0037214450347887 |
### Chart: PFO
| Category | |
|---|---|
| WT | 1.0 |
| brnQ2/Q3DKO | 0.7628611691076624 |
| brnQ1/Q3DKO | 0.5948564492385708 |
| brnQ1/Q2DKO | 0.9646540099053279 |
| WT | 1.0 |
| brnQ2/Q3DKO | 0.6819594946256422 |
| brnQ1/Q3DKO | 0.6672407048461588 |
| brnQ1/Q2DKO | 1.1307472416061566 |Figure S7. Comparison of Western blot intensities as determined by Image J for wild type (WT) and double null mutant strains. (A) PLC, (B) PFO. Shown are the mean values from three independent experiments. The error bars indicate the S.D. There were no significant differences (P>0.05) for any strains compared to WT.

## Slide 8
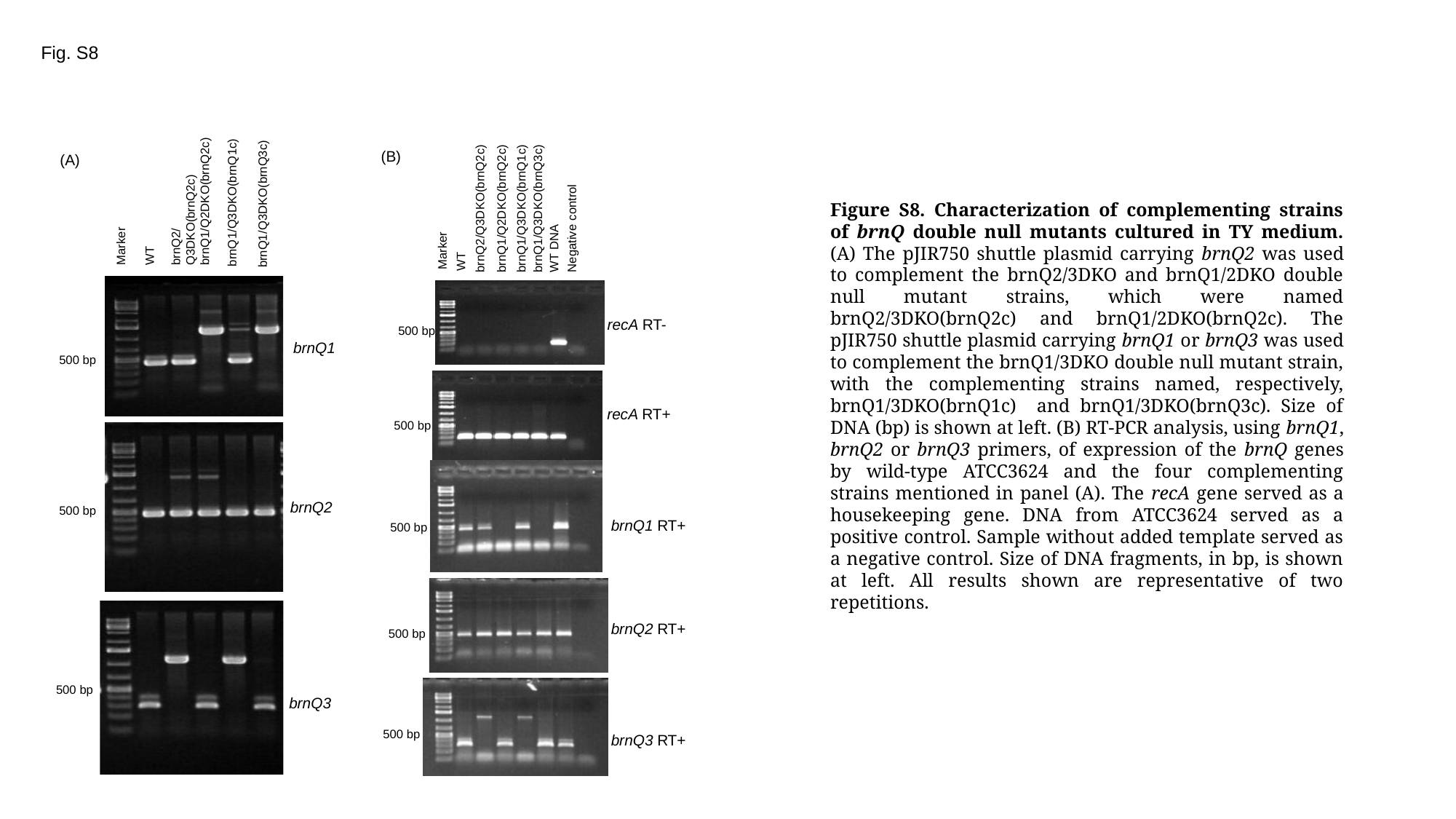

Fig. S8
brnQ1/Q3DKO(brnQ3c)
brnQ2/Q3DKO(brnQ2c)
brnQ1/Q2DKO(brnQ2c)
brnQ1/Q3DKO(brnQ1c)
brnQ1/Q2DKO(brnQ2c)
brnQ2/Q3DKO(brnQ2c)
brnQ1/Q3DKO(brnQ1c)
brnQ1/Q3DKO(brnQ3c)
(B)
(A)
Marker
WT
Marker
WT
WT DNA
Negative control
Figure S8. Characterization of complementing strains of brnQ double null mutants cultured in TY medium. (A) The pJIR750 shuttle plasmid carrying brnQ2 was used to complement the brnQ2/3DKO and brnQ1/2DKO double null mutant strains, which were named brnQ2/3DKO(brnQ2c) and brnQ1/2DKO(brnQ2c). The pJIR750 shuttle plasmid carrying brnQ1 or brnQ3 was used to complement the brnQ1/3DKO double null mutant strain, with the complementing strains named, respectively, brnQ1/3DKO(brnQ1c) and brnQ1/3DKO(brnQ3c). Size of DNA (bp) is shown at left. (B) RT-PCR analysis, using brnQ1, brnQ2 or brnQ3 primers, of expression of the brnQ genes by wild-type ATCC3624 and the four complementing strains mentioned in panel (A). The recA gene served as a housekeeping gene. DNA from ATCC3624 served as a positive control. Sample without added template served as a negative control. Size of DNA fragments, in bp, is shown at left. All results shown are representative of two repetitions.
recA RT-
500 bp
brnQ1
500 bp
recA RT+
500 bp
brnQ2
500 bp
brnQ1 RT+
500 bp
brnQ2 RT+
500 bp
500 bp
brnQ3
500 bp
brnQ3 RT+

## Slide 9
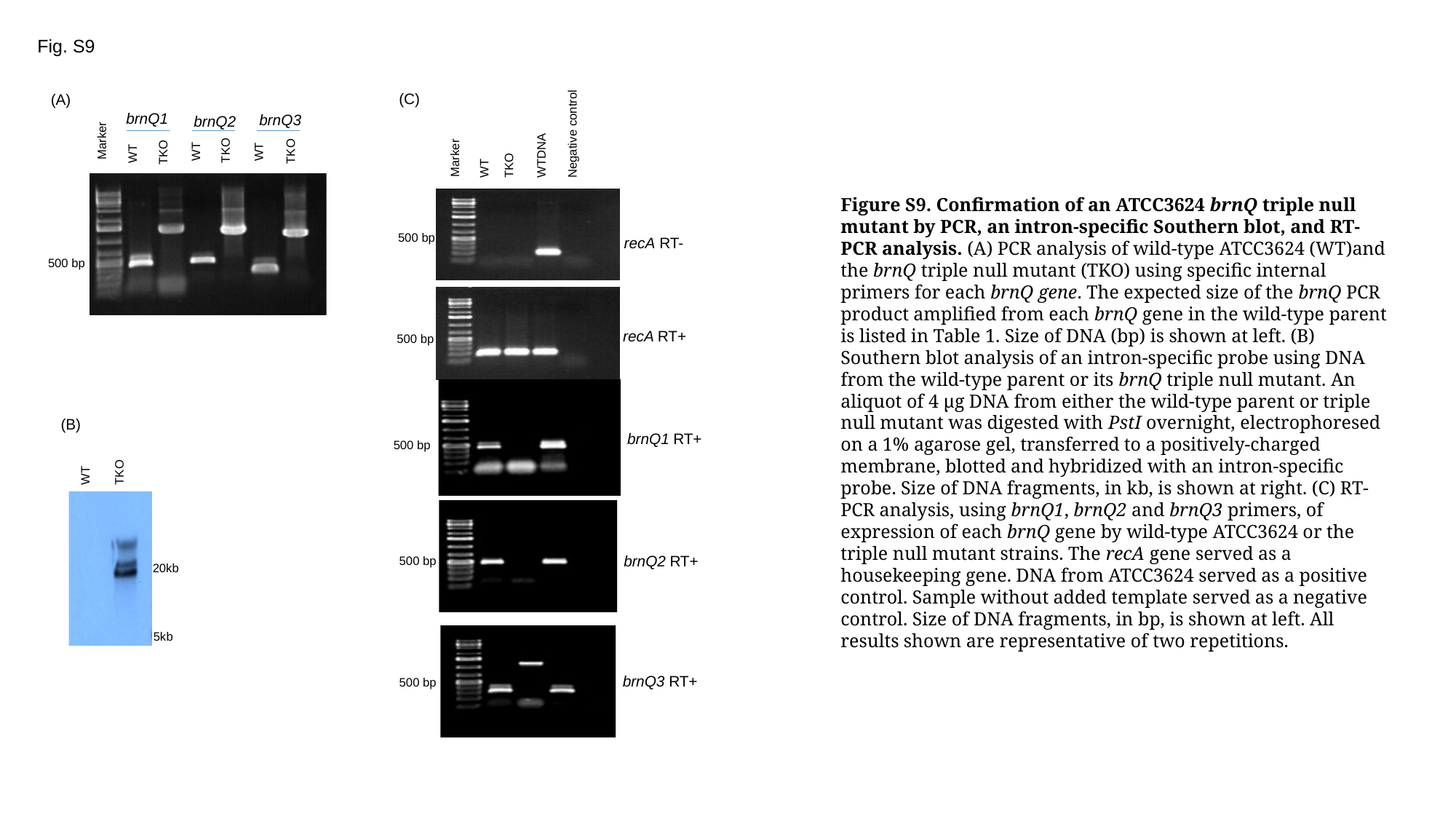

Fig. S9
Marker
WT
WT
WT
TKO
TKO
TKO
Marker
WT
TKO
WTDNA
Negative control
(C)
(A)
brnQ1
brnQ3
brnQ2
Figure S9. Confirmation of an ATCC3624 brnQ triple null mutant by PCR, an intron-specific Southern blot, and RT-PCR analysis. (A) PCR analysis of wild-type ATCC3624 (WT)and the brnQ triple null mutant (TKO) using specific internal primers for each brnQ gene. The expected size of the brnQ PCR product amplified from each brnQ gene in the wild-type parent is listed in Table 1. Size of DNA (bp) is shown at left. (B) Southern blot analysis of an intron-specific probe using DNA from the wild-type parent or its brnQ triple null mutant. An aliquot of 4 µg DNA from either the wild-type parent or triple null mutant was digested with PstI overnight, electrophoresed on a 1% agarose gel, transferred to a positively-charged membrane, blotted and hybridized with an intron-specific probe. Size of DNA fragments, in kb, is shown at right. (C) RT-PCR analysis, using brnQ1, brnQ2 and brnQ3 primers, of expression of each brnQ gene by wild-type ATCC3624 or the triple null mutant strains. The recA gene served as a housekeeping gene. DNA from ATCC3624 served as a positive control. Sample without added template served as a negative control. Size of DNA fragments, in bp, is shown at left. All results shown are representative of two repetitions.
500 bp
recA RT-
500 bp
recA RT+
500 bp
WT
TKO
(B)
brnQ1 RT+
500 bp
20kb
5kb
brnQ2 RT+
500 bp
brnQ3 RT+
500 bp

## Slide 10
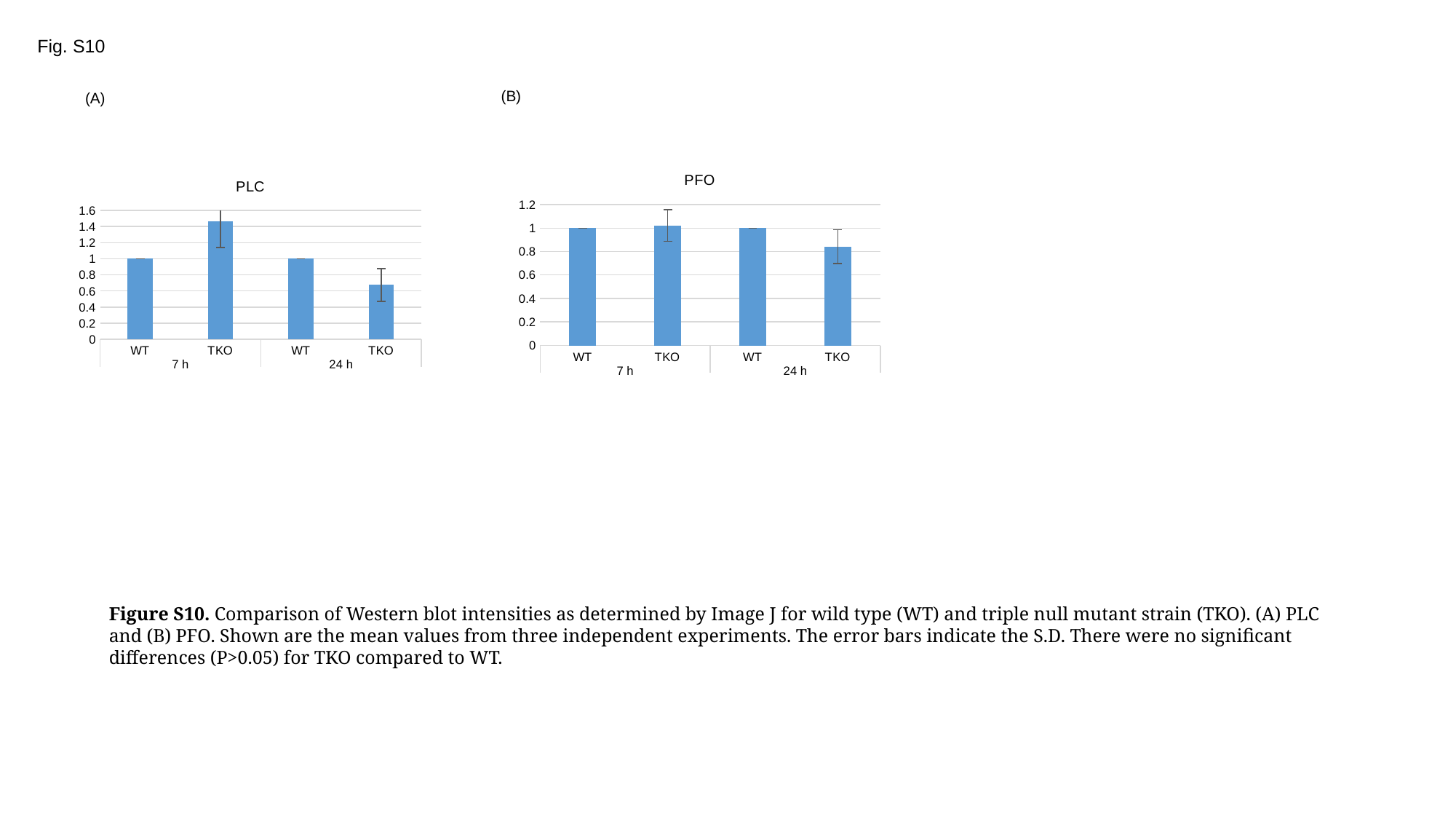

Fig. S10
(B)
(A)
### Chart: PFO
| Category | |
|---|---|
| WT | 1.0 |
| TKO | 1.021996706699237 |
| WT | 1.0 |
| TKO | 0.8424293867358671 |
### Chart: PLC
| Category | |
|---|---|
| WT | 1.0 |
| TKO | 1.4686460366823775 |
| WT | 1.0 |
| TKO | 0.6741816249052363 |Figure S10. Comparison of Western blot intensities as determined by Image J for wild type (WT) and triple null mutant strain (TKO). (A) PLC and (B) PFO. Shown are the mean values from three independent experiments. The error bars indicate the S.D. There were no significant differences (P>0.05) for TKO compared to WT.
